# Geometrically Induced Selectivity and Unidirectional Electroosmosis in Uncharged Nanopores

**DOI:** 10.1021/acsnano.1c03017

**Published:** 2022-05-19

**Authors:** Giovanni Di Muccio, Blasco Morozzo della Rocca, Mauro Chinappi

**Affiliations:** † Dipartimento di Ingegneria Industriale, Università di Roma Tor Vergata, Via del Politecnico 1, 00133, Rome, Italy; ‡ Dipartimento di Biologia, Università di Roma Tor Vergata, Via della Ricerca Scientifica 1, 00133, Rome, Italy

**Keywords:** electroosmosis, nanofluidics, induced charge, surface patterning, biological nanopores

## Abstract

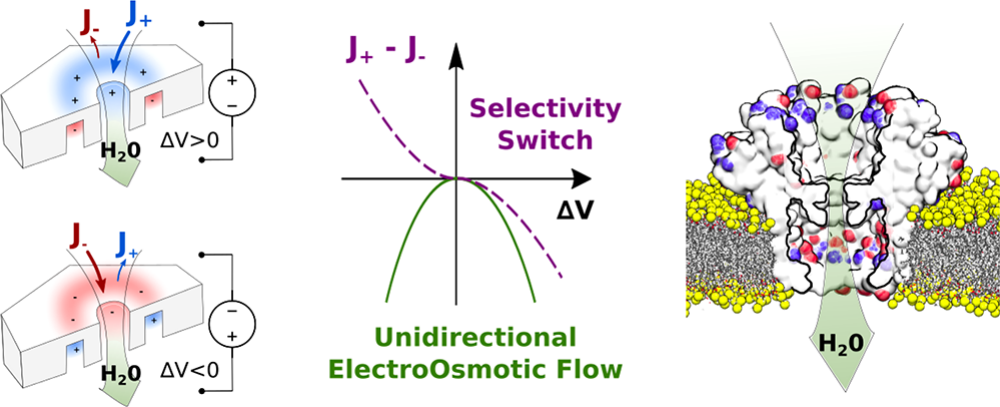

Selectivity toward positive and negative ions in nanopores is often associated with electroosmotic flow, the control of which is pivotal in several micro-nanofluidic technologies. Selectivity is traditionally understood to be a consequence of surface charges that alter the ion distribution in the pore lumen. Here we present a purely geometrical mechanism to induce ionic selectivity and electroosmotic flow in uncharged nanopores, and we tested it *via* molecular dynamics simulations. Our approach exploits the accumulation of charges, driven by an external electric field, in a coaxial cavity that decorates the membrane close to the pore entrance. The selectivity was shown to depend on the applied voltage and becomes completely inverted when reversing the voltage. The simultaneous inversion of ionic selectivity and electric field direction causes a unidirectional electroosmotic flow. We developed a quantitatively accurate theoretical model for designing pore geometry to achieve the desired electroosmotic velocity. Finally, we show that unidirectional electroosmosis also occurs in much more complex scenarios, such as a biological pore whose structure presents a coaxial cavity surrounding the pore constriction as well as a complex surface charge pattern. The capability to induce ion selectivity without altering the pore lumen shape or the surface charge may be useful for a more flexible design of selective membranes.

The transport of ions, water, small molecules, and polymers through transmembrane protein channels plays a fundamental role in sustaining cellular life, and it is drawing increasing attention thanks to the recent progress of nanofluidic technology.^1^ High cation or anion selectivity,^2^ diode-like current rectification,^3,4^ different gating mechanisms,^5−8^ surprisingly large flow rates,^9−12^ and other unexpected and *exotic* fluid phenomena at the nanoscale were unveiled in the last two decades.^13^ This fostered the development of technological applications based on either biological or synthetic nanopores, such as single-molecule nanopore sensing,^14,15^ blue energy harvesting,^16,17^ and high-throughput biomimetic filters.^18^

The coupling of the extreme fluid confinement, geometrical shape, and interfacial physicochemical properties leads to nontrivial electrohydrodynamic phenomena in nanofluidic systems. For example, cation or anion selectivity in nanopores is traditionally understood to be a consequence of charges present on the pore wall. Indeed, the electrolyte solution in contact with a charged surface forms an oppositely charged diffused layer, known as the Debye layer, at the solid–liquid interface.^19^ Due to the high surface-to-volume ratio, the Debye layer often occupies a non-negligible part of the lumen of charged nanopores. When a voltage is applied across the pore, the total electric current will be mostly formed by the predominant mobile charges (cations or anions) present in the Debye layer, resulting in a selective ionic transport. Moreover, the Coulombic force acting on the net charge of the Debye layer results in a force on the solvent that generates a fluid motion, usually indicated as electroosmotic flow (EOF). EOF plays a relevant role in nanopore sensing technology since it can compete or cooperate with electrophoresis and dielectrophoretic forces acting on the analyte,^20,21^ and it can be exploited to capture molecules independently of their charge.^22,23^

Many studies aimed at tuning ionic selectivity and EOF involve the chemical modification of the pore to introduce surface charges,^24−26^ but other mechanisms have been exploited. An example is provided by externally gated nanopores, where the pore surface charge is controlled *via* additional electrodes^27−32^ applied to the membrane substrate. External gating allows achieving good control of the pore selectivity, although the complex fabrication *de facto* limits its application for pores of nanometer or sub-nanometer diameter. Another strategy that can be employed to tune pore selectivity exploits induced-charge electrokinetic (ICEK) phenomena. Differently from externally gated selectivity control, in ICEK the same external electric field that drives the ions through the pore also polarizes the solid membrane, inducing a surface charge that, in turn, alters the Debye layer in the nanochannel and, hence, the selectivity and the EOF.^33,34^ A core ingredient to generate a net EOF by ICEK is the presence of some asymmetries in the system that give rise to inhomogeneities of ionic density distributions along the pore in response to the applied voltage. In the nanopore realm, this asymmetry is often introduced in the pore geometry (*e*.*g*., conical pores^34^) or imposing salt gradients through the membrane.^35^

Here, we propose a mechanism to induce a voltage-dependent ionic selectivity and EOF in uncharged cylindrical nanopores by taking advantage of geometrical asymmetries of the membrane without any external voltage-gating control, salt gradient, or chemical modification of the pore surface. Our system, Figure 1a,b, exploits the accumulation of charge between the pore lumen and a coaxially surrounding cavity. The induced selectivity is completely inverted by reverting the applied electric field. The concurrent inversion of ionic selectivity and applied voltage generates a unidirectional EOF, independently of the applied voltage polarity. Since the same electrical field that induces the pore selectivity is also responsible for the ion motion, the mechanism we propose can be included in the broad class of ICEK phenomena. We developed a theory, based on a continuum electro-hydrodynamical description, to assess the dependence of selectivity and EOF from applied voltage Δ*V* and pore geometry.

**Figure 1:**
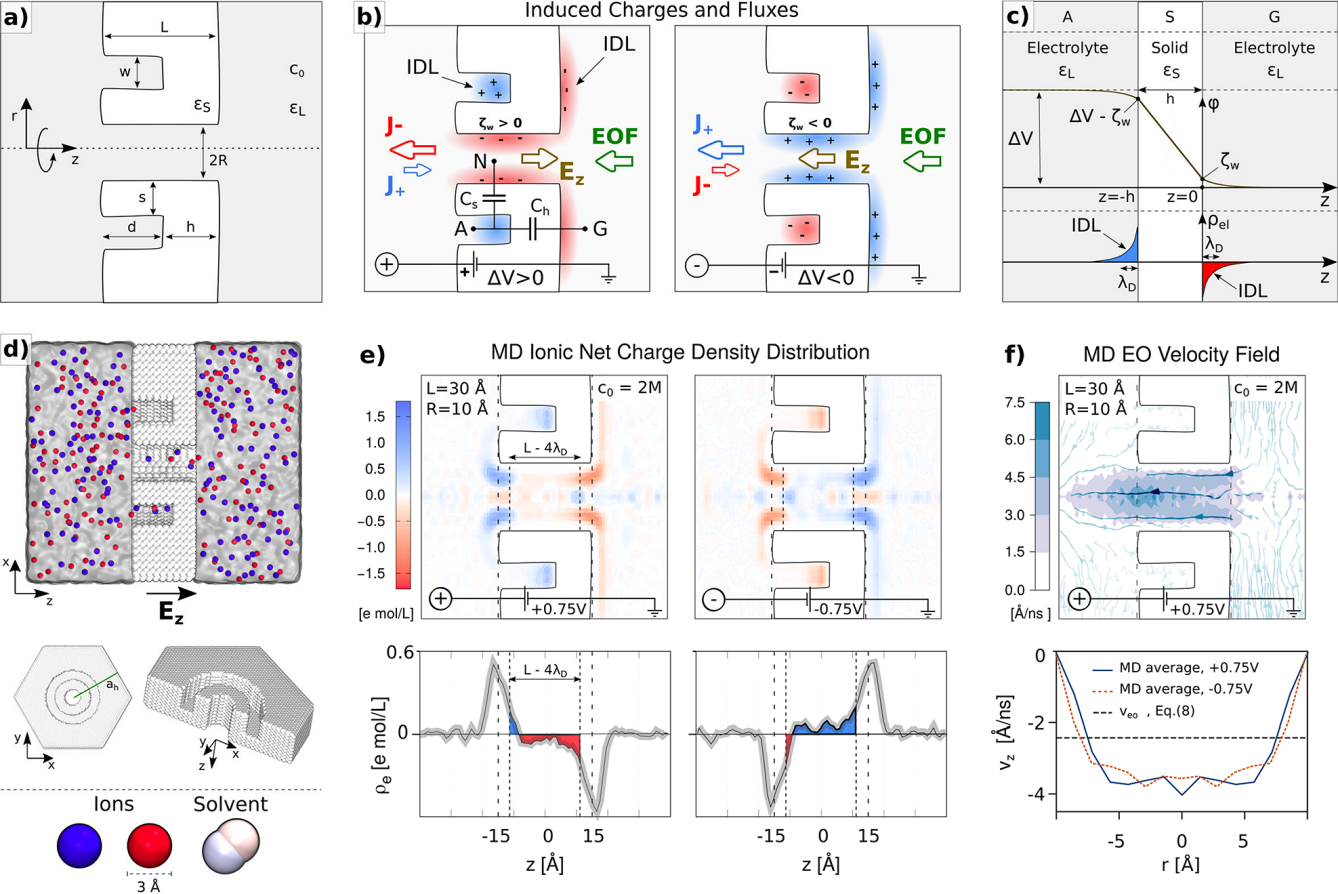
Geometrically induced selectivity switch. (a) Geometry of the system. A nanopore of radius *R* is drilled through a membrane of thickness *L*. The channel is surrounded by a coaxial cavity of width *w* and depth *d* = *L* – *h*, at a distance *s* from the nanopore wall. (b) Working principle. An external applied voltage Δ*V* gives rise to induced Debye layers (IDLs) at the solid–liquid interfaces, the polarity of which depends on the voltage sign. Meanwhile, the electric field *E*_*z*_ drives the ions through the nanopore. The presence of a charged IDL inside the nanopore results in a selective ionic transport (*J*_+_ ≠ *J*_–_), causing an electroosmotic flow (EOF). Since both the electric field *E*_*z*_ and the selectivity depend on the applied voltage polarity, the EOF (green arrow) is always oriented in the same direction. (c) Planar electrolytic capacitance. An infinite neutral membrane separates two reservoirs filled by the same electrolyte solution. When a voltage Δ*V* is imposed across the membrane, surface electric potentials ±ζ_w_ arise at the solid–liquid interfaces and charges are accumulated in the IDLs (blue and red areas), whose characteristic size is the Debye length λ_D_. (d) Molecular dynamics setup and tilted views of the membrane. White spheres represent the solid membrane atoms, blue and red ones are the positive and negative ions, and the transparent gray background is the solvent, composed of dipolar diatomic molecules, shown at the bottom. (e) Charge distribution from MD at Δ*V* = ±0.75 V, with *c*_0_ = 2 M salt concentration. The bottom plots represent the average net charge density in cylindrical sections of radius *R* = 10 Å along the pore axis. Confidence intervals, calculated using a block average with each block corresponding to 10 ns, are reported in shaded gray. (f) Electroosmotic velocity field from MD at Δ*V* = +0.75 V. Bottom panel represents the MD average velocity profile (*v*_*z*_ component) inside the pore (|*z*| < *L*/2 – 2λ_D_) at Δ*V* = ±0.75 V. The dashed line represents the model prediction, eq 8. MD distributions and fluxes are averaged over an 800 ns MD trajectory (16 000 frames); see Methods.

As a proof of principle, we set up molecular dynamics (MD) simulations of a model system composed of an uncharged solid-state nanopore surrounded by a coaxial cavity, Figure 1a,d. Our MD results show that the EOF depends quadratically on Δ*V*, in agreement with the theory. We also explored more complex scenarios where a surface charge is present at the pore wall to understand in which conditions the geometrically induced EOF is predominant with respect to EOF due to fixed surface charge. We finally show that selectivity switch and unidirectional EOF may also occur for the CsgG bacterial amyloid secretion channel,^36,37^ a protein pore employed in a commercial nanopore sequencing device.^38^ CsgG has a coaxial cavity like our simplified model and, in addition, presents a complex surface charge pattern, as usual for biopores.

## Results and Discussion

### Geometrically Induced Selectivity Switch: Working Principle and MD Simulations

Let us consider the system represented in Figure 1a, composed of a solid insulating membrane (white) of thickness *L* with a cylindrical nanopore of radius *R*, surrounded by a coaxial cavity of width *w* and depth *d* = *L* – *h*, at a distance *s* from the nanopore wall. The membrane (relative permittivity ε_S_) is immersed in 1:1 electrolyte solution (gray background) with relative permittivity ε_L_ and oppositely charged ions with the same ion mobility μ_±_ = μ. The pore is completely uncharged, so equilibrium (no applied voltage) ionic concentrations *c*_+_ and *c*_–_ are homogeneous everywhere and equal to the bulk value *c*_0_. When a voltage Δ*V* is applied across the nanopore, two main effects occur, as sketched in Figure 1b: (i) ions flow through the pore lumen (*J*_+_ and *J*_–_ arrows) and (ii) induced Debye layers (IDLs) form at the solid walls (blue and red charged clouds), depending on the voltage polarity. The presence of the cavity affects the IDL shape, resulting in an accumulation of charges across the cavity and the nanopore lumen, whose signs depend on the voltage polarity; see Figure 1b. The broken electroneutrality inside the pore results in ionic selectivity (anionic and cationic currents are different) and EOF.

In order to determine the dependence of the pore selectivity on the applied voltage Δ*V* we reasoned as follows. As a first approximation, electrophoretic ionic fluxes are proportional to the concentration and mobility of each species,^19^**J**_±_ = ±μ*c*_±_**E**, with **E** the driving electric field. We use the difference between the cation and anion fluxes as a measure of the ionic selectivity:
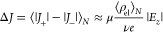
1with ρ_el_ = *νe*(*c*_+_ – *c*_–_) the net charge density, *v* the valence of the ions, and *e* the elementary charge, and where ⟨..⟩_*N*_ denotes the volumetric average inside the nanopore. So, selectivity depends on the sign of the charge of the IDL inside the nanopore lumen.

To quantify the IDL in the nanopore, we focus on the positive voltage case of Figure 1b, left side. A potential difference is present between the lateral cavity (point A at potential Δ*V*) and the right reservoir of the membrane (point G, grounded) and between the cavity and the pore lumen (point N).

The planar membrane solution description is instrumental to understanding the IDL dependence on voltage, Figure 1c. In the right reservoir (G), due to the potential difference (ζ_w_) between the bulk and the wall, negative ions accumulate close to the membrane surface, red area. Similarly, positive ions accumulate on the left side (A), blue area. Inside the membrane the electric potential ϕ(*z*) decays linearly. ζ_w_ is proportional to the applied voltage Δ*V*; see Supplementary Note S1 and Supplementary Figure S1 for details. Since the accumulated charge in the IDL is also linear in Δ*V*, the process can be described as a capacitance between A and G. Extending this reasoning to our nanopore system, the charge accumulation between the lateral cavity (point A) and the nanopore lumen (point N) can be modeled as a capacitance. Actually, the potential difference between the lateral cavity and the nanopore lumen is a function of the *z* coordinates since the potential inside the pore lumen varies along the nanopore axis. Nevertheless, in a quasi-1D approximation (see Supplementary Note S1), the total charge *q*_N_ inside the nanopore is still proportional to the applied voltage, *i*.*e*., *q*_N_ = −*C*_s_Δ*V*, with
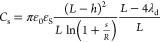
2an equivalent capacitance between the cavity and the pore that depends only on geometrical parameters. Therefore, the average net charge density inside the nanopore is
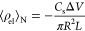
3and, consequently, the ionic selectivity, eq 1, reads
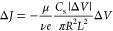
4Equation 4 shows that selectivity reverts when inverting the applied voltage Δ*V*, and its magnitude depends on Δ*V* quadratically.

We tested the validity of the above analytical model at the nanoscale by using all-atom MD simulations. To get rid of any asymmetries of the electrolyte that may potentially give rise to competing selectivity of the nanopore (*e*.*g*., differences between ion mobilities, different hydration shells around cations and anions, preferential interaction of one ion with the solid), we built a custom symmetric model for the electrolyte solution. In particular, we considered two monovalent ionic species with the same mass dissolved in a liquid composed of diatomic dipolar molecules. The membrane is composed of neutral atoms. All the atoms have the same van der Waals radius, and the volume of the solvent molecule is similar to water; see Methods for details and Supplementary Figures S4–S9 for a characterization of the fluid in terms of phase diagram, relative electrical permittivity, wetting, ion mobility, and viscosity.

**Figure 2:**
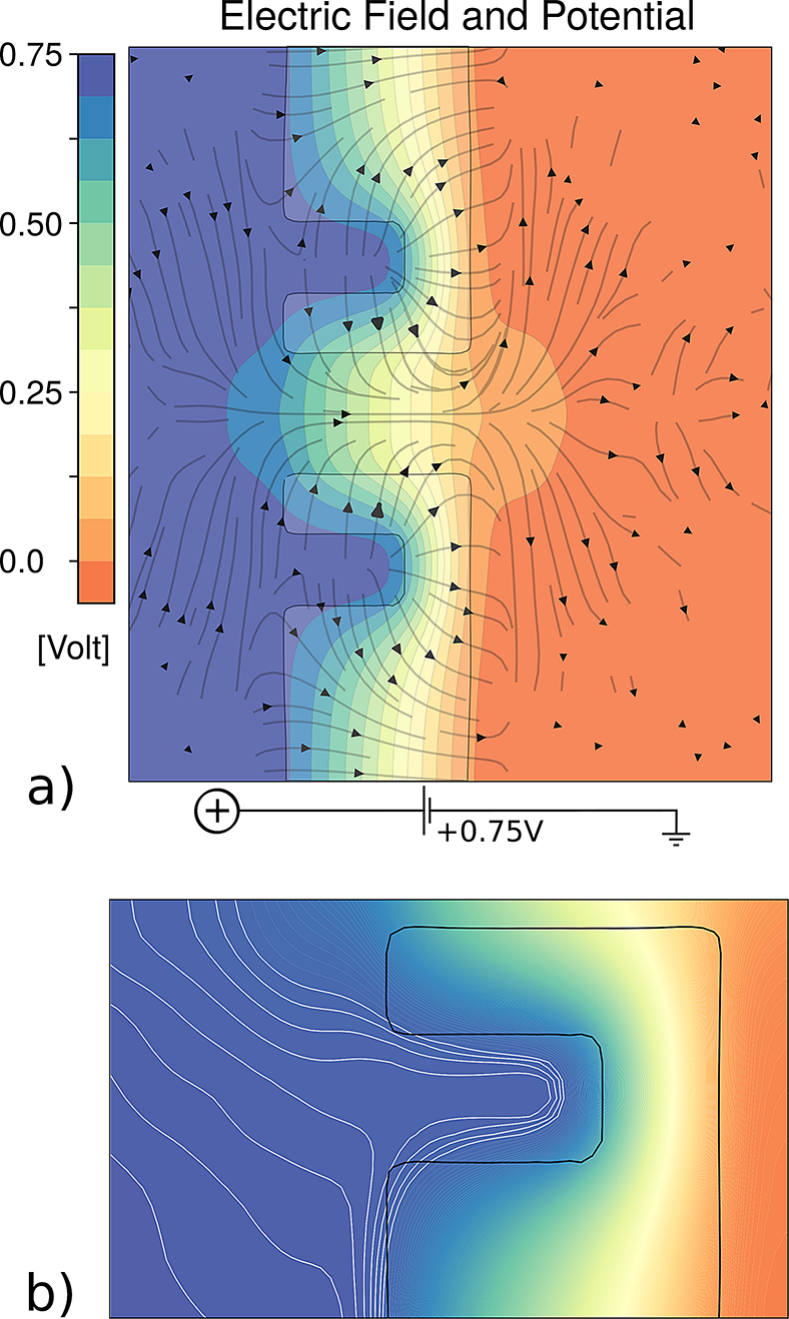
(a) Electric potential map. The black arrowed lines represent the electric field **E**(*r*, *z*) = −∇*V*. We filtered out the lines where |*E*(*r*, *z*) | < 13% of the maximum intensity. The potential map is averaged over an 800 ns MD trajectory (16 000 frames); see Methods and refer to the MD simulation of the 2 M system shown in Figure 1d–f, with *R* = 10 Å, *L* = 30 Å, *h* = 10 Å, *s* = 9 Å, and *w* = 12 Å at Δ*V* = +0.75 V transmembrane applied bias. (b) Zoom-in on the cavity. The isolines roughly follow the solid walls, indicating the presence of the induced Debye layer inside the cavity. Selected isolines in the left reservoir are highlighted in white for clarity.

We first studied a system with pore length *L* = 30 Å, pore radius *R* = 10 Å, cavity width *w* = 12 Å, and depth *d* = 10 Å at distance *s* = 9 Å, for a 2 M solution Figure 1d. Ionic net charge densities are reported in Figure 1e for positive Δ*V* = +0.75 V and negative applied voltage Δ*V* = −0.75 V, showing the formation of IDLs. It is apparent that when a positive voltage is applied, positive charges are accumulated inside the cavity and a corresponding negative IDL arises along the pore. The opposite happens for negative bias. The characteristic length scale of the IDL appears to be, as expected, on the order of the Debye length of the electrolyte solution, λ_D_ ≃ 2 Å, in this case. Moreover, liquid velocity profiles show an EOF directed from right to left for both positive and negative voltages, Figure 1f. The MD simulations revealed additional features of the charge distributions, such as the two opposite charge density peaks appearing at the nanopore entrance and discontinuous patterns along the pore axis. Nevertheless, the overall IDL formation mechanism proposed in Figure 1b is confirmed: when changing the applied voltage, the selectivity of the pore switches from cations to anions. The electric potential estimated from MD simulations (Figure 2) further confirms the trend of the voltage drops schematically described in our model. The electric potential decreases quite linearly along the pore, while a large part of the cavity is approximately isopotential with respect to the left reservoir (Δ*V* = +0.75 V). More in detail, the isolines follow the wall surface inside the cavity, indicating that the IDL contours the wall profile, Figure 2b.

### Parabolic Electroosmosis

**Figure 3:**
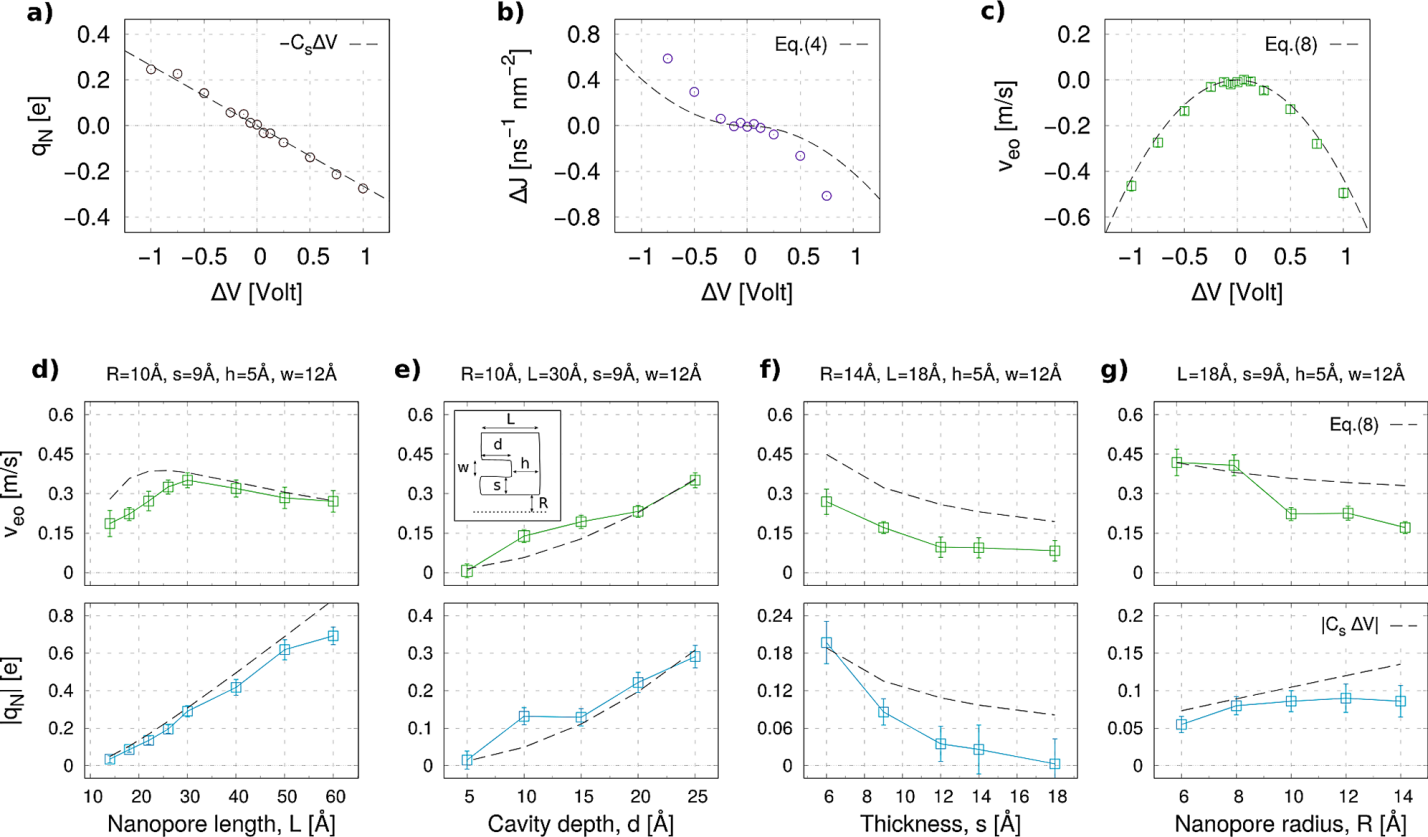
Electrohydrodynamic fluxes and charges in the nanopore. (a–c) Charge in the pore (*q*_N_), selectivity (Δ*J*), and average EO velocity (*v*_eo_) from MD simulation of the 2 M system shown in Figure 1d–f, with *R* = 10 Å, *L* = 30 Å, *h* = 10 Å, *s* = 9 Å, and *w* = 12 Å. Dashed lines refer to the analytical model described in the text. (d–g) Electroosmotic velocity and total charge in the pore as a function of (d) pore length *L*, (e) depth of the cavity *d*, (f) thickness *s*, and (g) pore radius *R*. Analytical model results are shown as dashed lines, and MD data as colored squares. Each error bar represents the standard error obtained from an 800 ns MD trajectory (16 000 frames). Inset in (e) recalls the geometric parameters of our model.

As anticipated in the previous section, a major consequence of the selectivity switch is that the EOF is always negative in our framework (Figure 1b), *i*.*e*., directed from the right to the left side of the membrane, for both positive and negative voltages. An analytical insight into the dependence of EOF on Δ*V* can be derived using a continuum electrohydrodynamics approach based on the Poisson–Nernst–Planck and Navier–Stokes (PNP–NS) equations.^19^ The PNP–NS system is derived under several assumptions that are not always respected at the nanoscale, such as the continuum assumption. Moreover, in order to get a practical analytical solution, we needed to rely on several additional hypotheses, such as dilute solution limit and homogeneous mobility. A discussion of these hypotheses and their implications is reported in Supplementary Note S2. For λ_D_ ≪*R* (no Debye layer overlap), PNP–NS predicts that the electroosmotic volumetric flow rate (*Q*_eo_) through a cylindrical channel of radius *R* and length *L* can be written as
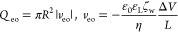
5with ε_L_ and η are the relative permittivity and viscosity of the electrolyte solution; ζ_w_ is the average surface electrokinetic potential^39^ and *v*_eo_ is the Helmholtz–Smoluchowski electroosmotic velocity, *i*.*e*., the velocity of the plug flow obtained when λ_D_ ≪ *R*.^40^ Note that, in this work, *v*_eo_ is positive if directed from left to right; see Figure 1a. In this framework, the net charge density ρ_el_ and, hence, the total charge *q*_N_ inside the nanopore are a function of ζ_w_:
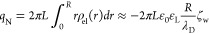
6where in the rightmost term we considered that for *R* ≫ λ_D_ the charge in the pore can be approximated as the product of pore surface 2π*RL* times the surface charge of a planar Debye layer ε_0_ε_L_ζ_w_/λ_D_.^19^ Thus, ζ_w_ is proportional to *q*_N_ and, for eq 3, to Δ*V*. Combining eqs 6 and 3 we get
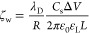
7that, when introduced into eq 5, leads to the parabolic expression for the EOF velocity:
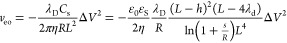
8Equations 5–8 are strictly valid only for λ_D_ ≪ *R*, and therefore, in principle, accurate quantitative predictions cannot be expected. Nevertheless, for the pore in Figure 1d–f (*L* = 30 Å and *R* = 10 Å) the model predictions are in very good agreement with MD data. The capacitance *C*_s_, eq 2, well predicts the dependence of net pore charge *q*_N_ on Δ*V*, dashed line in Figure 3a. The MD selectivity Δ*J*, computed from the ionic currents shown in Supplementary Figure S10, is reported in Figure 3b, confirming the selectivity switch predicted by eq 4 of our model. The higher MD values may be explained by the convective contribution to ion transport that is not included in eq 1. Indeed, since the EOF is directed as the dominant ionic flow, it always results in an increase of selectivity. Finally, eq 8 gives an excellent quantitative estimation of the average electroosmotic velocity, *v*_eo_ = *Q*_eo_/π*R*^2^, with *Q*_eo_ computed from MD simulations, Figure 3c.

### Effect of Geometric Parameters

To verify the robustness of the observed phenomenon and the accuracy of the proposed quantitative model, we performed a second set of MD simulations focusing on the role of geometrical parameters. Each set of simulations is performed at Δ*V* = +0.75 V, by varying one single geometrical parameter while keeping fixed all the others. Results are reported in Figure 3d–g, with a sketch of the geometry reported in the inset of Figure 3e. The electroosmotic velocity |*v*_eo_| is reported on the top panels, while the total accumulated charge inside the nanopore |*q*_N_| is shown in the bottom ones. We observe induced charge accumulation inside the pore and a concomitant EOF in all cases. The general trends predicted by our model are in good agreement with the simulations. The quasi-1D capacitance model, eq 2, predicts the MD data within two error bars for almost all cases. The analytical *v*_eo_, eq 8, better matches the MD data for longer pores (*L* > 30 Å), while it slightly overestimates the flow rates for the shorter ones; see Figure 3d. Anyhow, the model correctly indicates that the dependence on *L* is nonmonotonic; this is due to the competing effect between the driving electric field *E*_*z*_ = Δ*V*/*L*, which decreases with *L*, and the induced capacitance *C*_s_, eq 2, that increases with *L*. The induced charge effect and EOF increase with the cavity depth *d* = *L* – *h*, Figure 3e, consistent with the increase of the voltage drop between the pore lumen and the deeper portion of the cavity; see the quasi-1D pore capacitance model in Supplementary Note S1 and the electric potential maps in Supplementary Figure S11. The geometrically induced selectivity vanishes for *d* → 0, as trivially expected since the system becomes symmetric. The MD data of Figure 3e refer to a pore with *L* = 30 Å and, as for Figure 3d, are in quantitative agreement with the model. We also ran simulations for *L* = 18 Å, at different thickness *s* and radius *R*. In both cases, the model overestimates *q*_N_ and *v*_eo_ although capturing the trends of the MD data; for example, for increasing *s* the lateral capacitance decreases and so do *q*_N_ and *v*_eo_. The apparent quantitative agreement for *R* < 10 Å could be more probably ascribed to fortuitous compensation of different sources of atomistic effects than to a correct description of such extremely confined conditions.

The geometrically induced selectivity and the unidirectional EOF are not limited to nanometer and sub-nanometer scale. Equation 8 allows quantifying EOF for pores of any size and can hence be employed for nanopore system design. As an example, in Supplementary Figure S12, we report *v*_eo_ for a water electrolyte solution through a silicon nitride pore of radius *R* = 20 nm. Such relatively large pores are widely used in experimental studies,^41,42^ and the required surface patterning can be achieved with well-established techniques.^43^Equation 8 indicates that as the system size increases, |*v*_eo_| decreases. This decrease can be partially compensated using materials with larger dielectric constants or increasing the Debye length, as both λ_D_ and ε_S_ appear in the eq 8 numerator, but with some caveats discussed in Supplementary Note S2. Briefly, for λ_D_, eq 8 can reasonably estimate the flux only until λ_D_/*R* ≪1 (no Debye layer overlap). Similarly, the low concentrations needed to achieve relatively large λ_D_ will result in a small number of ions in the nanopore, an occurrence that may lead to the failure of the PNP–NS model to yield quantitative predictions. For a pore of radius *R* = 20 nm, eq 8 indicates that a |*v*_eo_| ≃ 0.1 m/s can be obtained; see Supplementary Figure S12. This EOF can be in principle experimentally measured. A possible technique is the one proposed by Secchi *etal*.,^11^ where the velocity field far from the pore is measured following the trajectory of tracers. This approach allows measuring the flow only at a distance of a few μm but not close to the pore. Nevertheless, a |*v*_eo_| ≃ 0.1 m/s at the exit of a pore of *R* = 20 nm would result in a velocity of magnitude *v* ≈ 0.4 × 10^–4^ m/s at a distance of 1 μm from the pore (fluid velocity scales as 1/*r*^2^, with *r* the distance from the pore). This value appears to be within reach of the proposed experimental technique^11^ and can be generated under an applied voltage of 1 ≤ Δ*V* ≤ 2 V, depending on the salt concentration (0.2 or 0.02 M) and the geometry; see Supplementary Figure S12.

Another approach to experimentally validate our results is to infer the EOF from its effect on the capture of nanoparticles by a nanopore. Indeed, the capture rate is ruled by the competition/cooperation of different effects, the most relevant being electrophoresis, electroosmosis, and dielectrophoresis.^21−23^ Analytical expressions for the capture rate have been recently proposed,^21^ and, in principle, they allow directly to relate EOF and capture rate, if pore and particle geometry, charge, and dielectric properties are known. Due to the difficulties in modeling pore entrance effects, quantitatively accurate estimations of EOF are not expected; nevertheless, a clear indication of the EOF direction and of the dependence of *v*_eo_ on Δ*V* should be achievable.

### Application to Weakly Charged Solid-State Nanopores

The theoretical model we developed is valid for neutral pores, *i*.*e*., no intrinsic surface charge is present at the pore walls. For silicon nitride, a widely used material for solid-state nanopores, the zero-charge condition is achieved at pH ≃ 4.1.^44,45,47^ Moreover, coatings can be used to alter the zero-charge pH, making it possible to get weakly charged pores (a few mC/m^2^) for wide ranges of pH.^45^ Instead, for HfO_2_, another material used for nanopores,^48^ the zero-charge pH is ≃7.5.^49^ A partial list of materials and conditions where the nanopore surface is neutral and, hence, geometrically induced selectivity and EOF can be effectively employed is reported in Supplementary Table S1.

**Figure 4:**
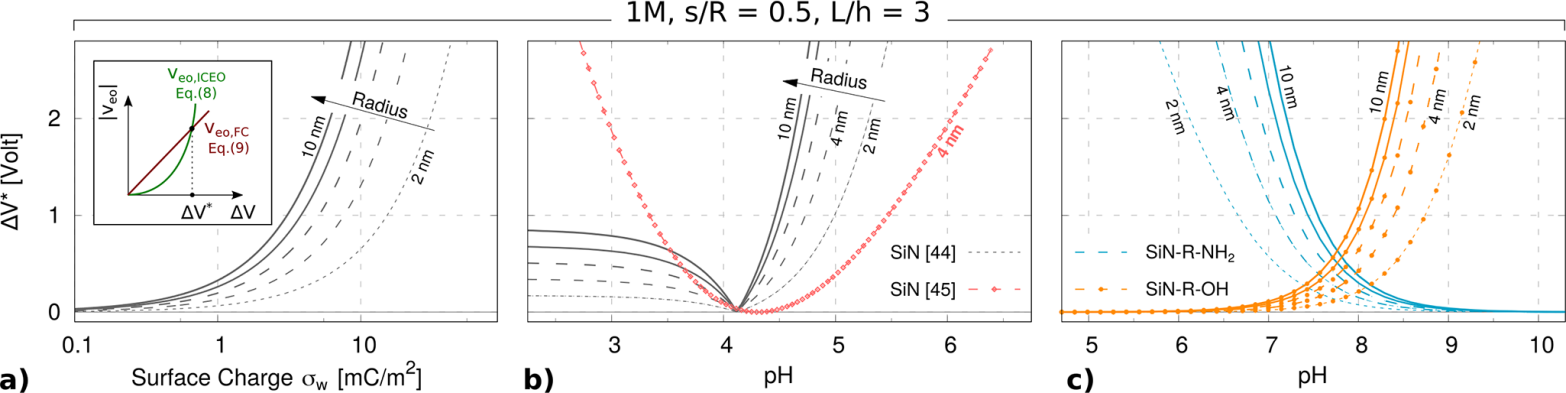
Threshold voltage ΔV* in the presence of a fixed surface charge. Threshold voltage ΔV* is defined in eq 10 as the voltage where the magnitude of fixed charge EO velocity, eq 9, and induced charge EO velocity, eq 8, are equal, as sketched in the inset of panel (a). (a) ΔV* as a function of fixed surface charge σ_w_, for pores of increasing radius from *R* = 2 nm to *R* = 10 nm. (b) pH dependence of ΔV* for silicon nitride pores, for different radii. Experimental fit for σ_w_ = σ_w_(pH) dependency on pH was taken from Lin *etal*.^44^ (black curves) or Bandara *etal*.^45^ (red curve); see Methods. (c) pH dependence of ΔV* for surface-modified silicon nitride pores with amine (cyan) or hydroxyl (orange) moieties, σ_w_ = σ_w_(pH), taken from Bandara *etal*.;^45^ see Methods. Reported examples are with fixed ratios *L*/*h* = 3 and *s*/*R* = 0.5 at 1 M KCl.

The capability to control surface charge in solid-state pores naturally raises a question on the relative impact of EOF due to fixed surface charge and the geometrically induced mechanism presented in this work. As a first approximation, EO velocity due to fixed surface charge density σ_w_ can be expressed as
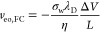
9which, in essence, is eq 5 with σ_w_ = ϵ_0_ϵ_L_ζ_w_/λ_D_; see Supplementary Note S2. Since *v*_eo,FC_ scales with Δ*V*, while geometrically induced electroosmotic velocity, eq 8, scales as Δ*V*^2^, at large enough Δ*V* the latter becomes dominant; see inset in Figure 4a. The magnitude of the threshold voltage ΔV* where the intensity of two contributions is equal can be obtained by combining eqs 9 and 8, resulting in
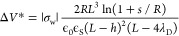
10ΔV* depends not only on geometrical parameters but also on surface charge σ_w_ and Debye length λ_D_, which, in turn, depends on pore material, pH, and ionic strength. As a first example, Figure 4a reports ΔV* as a function of σ_w_ in pores of radii between 2 and 10 nm. It is evident that, for σ_w_ < 5 mC/m^2^, ΔV* ≤ 2 V even for quite large nanopores (*R* = 10 nm), while ΔV* ≤ 0.5 V for the narrower one (*R* = 2 nm). Instead, Figure 4b shows ΔV* as a function of pH for bare SiN nanopores. We employed two analytical models describing σ_w_ as a function of pH,^44,45^ based on fitted experimental data; see Methods. For both of them, ΔV* is below 1 V in a relatively wide range of pH. Indeed, in bare SiN nanopores both silanol groups and amines are usually exposed on the surface,^44^ and σ_w_ changes sign around pH 4.1–4.3 (point of zero charge). By using surface modification, it is possible to keep a low σ_w_, and thus low ΔV*, for a wider range of pH, Figure 4c.^45^ In particular, for the reported SiN-R-OH-modified nanopore, with R alkane linker, the pore is essentially neutral for pH < 7. Conversely, the amine-modified SiN-R-NH_2_ nanopore is, in essence, neutral for pH > 8. 5. In these pH ranges, ΔV* < 150 mV for 10 nm radius pores and is even smaller for smaller radii.

The above arguments implicitly assume a superposition of effects; that is, the total EOF can be decomposed as the sum of fixed charge and induced charge contributions. This hypothesis is quite strong, so the estimation provided by eq 10 should be understood as a way to determine approximate voltage ranges where the intrinsic selectivity or the induced charge mechanism dominates the EOF. The above theoretical arguments are supported by MD simulations of a model pore (similar to the one shown in Figure 1), modified with a surface charge of σ_w_ = 2.5 or 5 mC/m^2^; see Supplementary Figure S13. For these two systems, MD simulations confirm that above the theoretical ΔV* the geometrically induced EOF dominates on the EOF due to fixed charges. The *v*_eo_ dependence on the voltage is still parabolic although shifted, in line with the superposition of effects hypothesis underlying eq 10.

### Effect of Asymmetric Electrolyte

**Figure 5:**
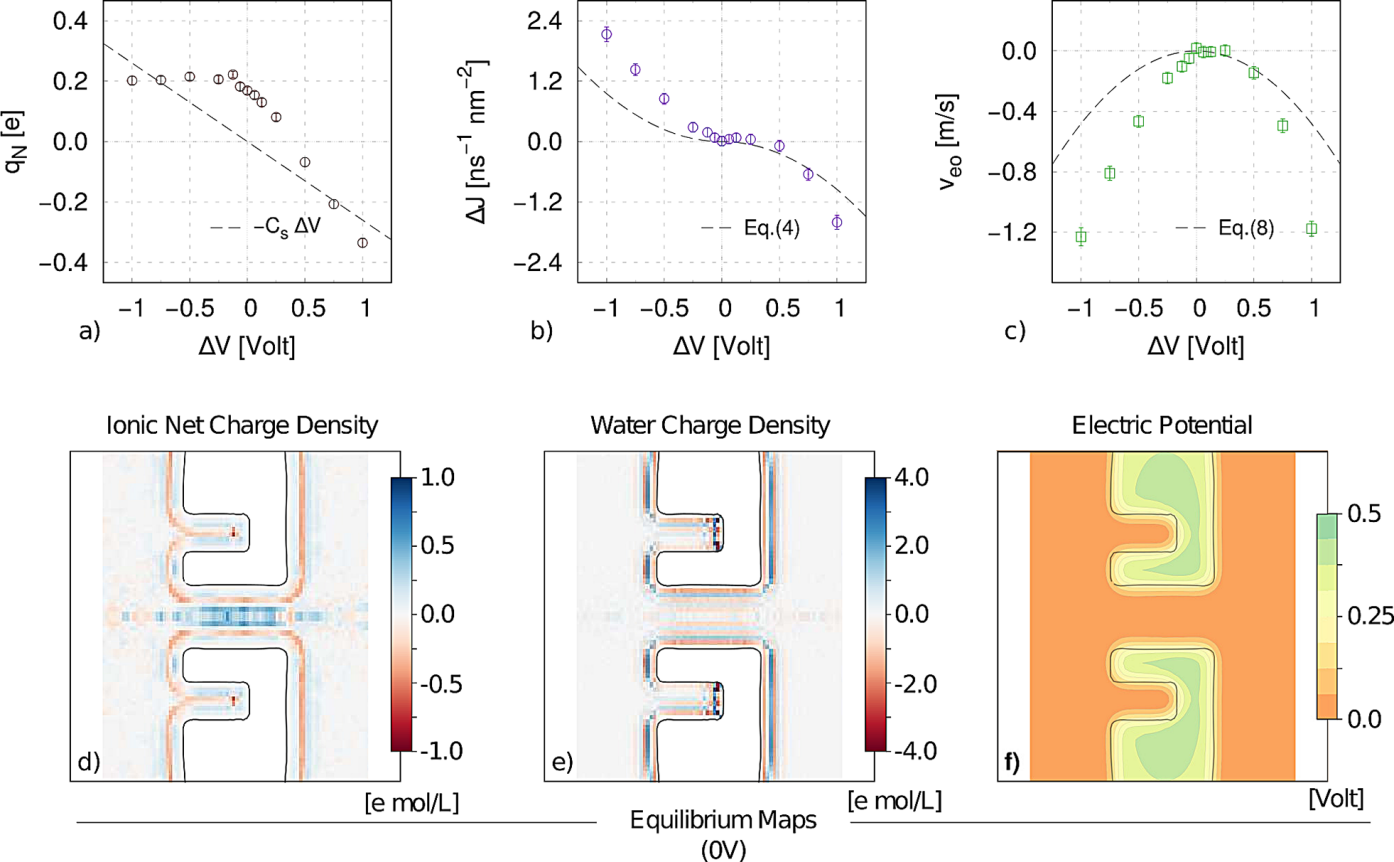
Effect of asymmetric electrolyte. (a–c) Charge in the pore *q*_N_, selectivity Δ*J*, and average EO velocity *v*_eo_ from MD simulation of a nanopore with *R* = 10 Å, *L* = 30 Å, *h* = 10 Å, *s* = 9 Å, and *w* = 12 Å (same as Figure 1d–f) in a 2 M KCl water solution (symbols). Gray dashed lines represent the theoretical predictions for a symmetric case, *i*.*e*., *q*_N_ = −*C*_s_Δ*V* for the nanopore charge and eqs 3–8 for Δ*J* and *v*_eo_. The other parameters used are μ = 1.0 × 10^3^ Å^2^/(V ns), λ_D_ = 2.1 Å, ε_S_ = 1, and η = 0.3 mPa s (TIP3P viscosity ≃ 1/3 experimental water^46^). (d–f) Ionic and water charge density and electric potential at equilibrium (Δ*V* = 0), showing the intrinsic polarization and layering at the solid–liquid interface, despite the zero charge of the solid membrane. The potential difference between the bulk liquid and the membrane interior is related to the presence of interfacial charge dipoles. MD distributions and fluxes are averaged over an 800 ns MD trajectory (16 000 frames). Errors are calculated using a block average protocol with a block length of 10 ns.

We then performed MD simulations of a nanopore system releasing one of the model hypotheses: the molecular symmetry of the electrolyte. Instead of using our custom perfectly symmetric electrolyte employed for the MD simulation data in Figures 1–3, in this section we used a 2 M KCl water solution. Now the mobilities of the two ions are different, as well as the structure of the first shell of water molecules around them. The overall behavior of the system is similar to the symmetric electrolyte case. In particular, a selectivity switch and a unidirectional EOF are observed; see Figure 5a–c. Some asymmetries are evident, as expected. At equilibrium, Δ*V* = 0, the system exhibits an intrinsic net positive charge accumulation inside the nanopore lumen (*q*_N_ ≃ 0.2*e*, Figure 5a,d), despite the zero surface charge of the solid. Indeed, the asymmetric electrolyte develops an equilibrium charge layering at the solid–liquid interface, Figure 5d. This is also evident from the peculiar orientation of the water molecules at the wall, forming surface dipoles, Figure 5e. The presence of interfacial dipoles generates an intrinsic polarization of the membrane and, hence, a nonzero surface potential, Figure 5f. The formation of a nonzero surface potential in uncharged nanopores due to electrolyte asymmetries was proposed by Dukhin *etal*.^50^ and later investigated by other authors.^51,52^ For instance, in Kim *etal*.^51^ it was shown that the different hydration forces among cations and anions lead to a slightly different equilibrium position of positive and negative charges (*i*.*e*., a charge layering) at the solid/liquid interface of uncharged hydrophobic nanopores. The charge layering results in a nonzero surface potential and EOF. A similar layering was also found in Mucha *etal*.^52^ at liquid/air interfaces.

Hence, for an asymmetric electrolyte, two effects rule the pore charge accumulation: the pore lumen’s equilibrium surface potential that leads to an intrinsic selectivity (cation, in the present case) and the induced charge mechanism due to the presence of the lateral cavity. We observe different behaviors under opposite Δ*V*; see Figure 5a–c. For Δ*V* < 0, the charge inside the nanopore, *q*_N_, remains relatively constant and the selectivity and EOF are both roughly proportional to Δ*V*. For Δ*V* > 0, instead, *q*_N_ decreases linearly with Δ*V*, and, coherently to the induced charge mechanism, the selectivity and EOF are quadratic. In such a complex scenario, the theoretical expressions derived for the perfectly symmetric case (dashed gray lines in Figure 5a–c) fall short in predicting quantitatively the selectivity and EOF intensity. Nevertheless, they still provide the order of magnitude of the effect.

### A Biological Example: The CsgG Nanopore

**Figure 6:**
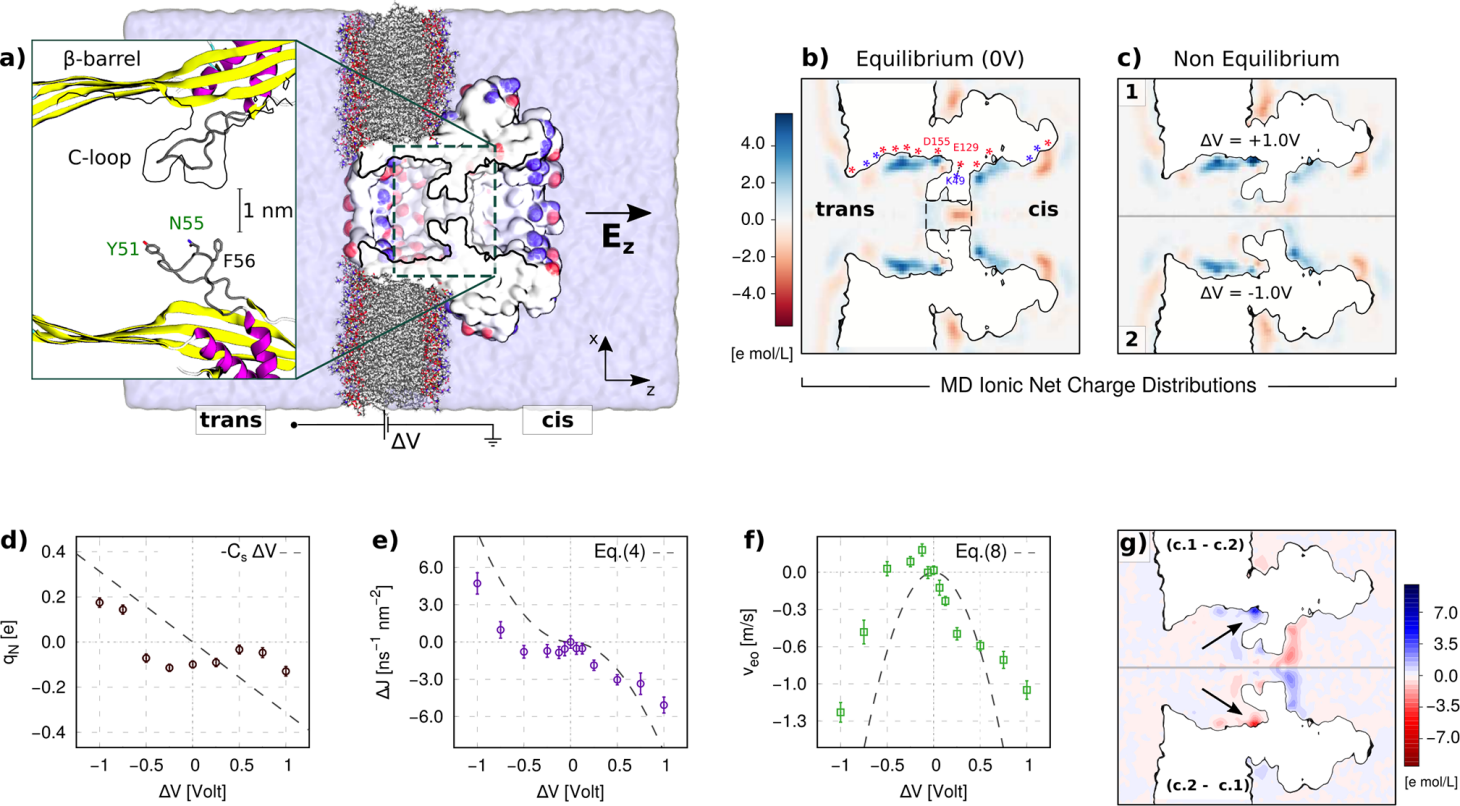
CsgG biological nanopore in 2 M KCl water solution. (a) MD setup. A volume rendering representation of the pore cross-section (white) embedded in a lipid membrane, with exposed charged residues colored (blue positive, red negative). Water and ions are omitted for clarity. The inset shows a zoom-in of the pore constriction with the cartoon representation of the secondary structure on the top side and licorice representation of the residues forming the constriction surface Y51 and N55 (hydrophilic, green labels) and F56 (hydrophobic, black label) on the bottom. (b) Equilibrium (Δ*V* = 0 V) and (c) nonequilibrium (Δ*V* = ±1 V) MD ionic net charge density distributions. The asterisks in (b) indicate the charged residues exposed toward the nanopore lumen. (d) Charge in the constriction, (e) selectivity, and (f) electroosmotic velocity as functions of the applied voltage Δ*V*. Dashed lines represent the theoretical prediction (*L* = 18 Å, *R* = 6 Å, *s* = 9 Å, *h* = 5 Å, and ε_S_ = 6). The other parameters for the solvent are the same as used in Figure 5. (g) Difference of the panels c.1 and c.2, pointing out the opposite charge accumulation inside the lateral cavity at opposite voltages Δ*V* = ±1 V. In panels (a)–(c) and (g) the black line delimiting the pore and the membrane is the water density contour level ρ = 0.5ρ_bulk_, with ρ_bulk_ being the bulk water density. Fluxes and maps are obtained from 280 ns MD production runs. All the trajectories are sampled every 20 ps and analyzed discarding the first 10 ns. Errors are calculated using a block average protocol with a block length of 10 ns.

We then verified if the geometrically induced selectivity switch and the unidirectional EOF also occur in more complex scenarios such as biological nanopores where articulate geometries and surface charge patterns are usually present. We selected as a possible candidate the curli specific gene G (CsgG) protein from *E. coli*. This pore is currently used in commercial devices for nanopore DNA sequencing.^38,53^ CsgG is a nonameric membrane protein, part of a transport machinery comprising at least seven proteins encoded by two operons^54^ that excrete functional amyloids,^55^ the curli proteins.^36,37^ The CsgG pore is constituted by two large vestibules on the cis and trans side connected by a constriction of diameter ≃1.2 nm, formed by the so-called C-loop, Figure 6a.

CsgG pore lumen is irregular, yet the shape of its constriction region resembles the cylindrical pore surrounded by a coaxial cavity, albeit being more complex. For example, the constriction region is not straight but has a cleft at about one-third of its length. The lateral cavity is formed between the transmembrane β-barrel and the C-loop (residues 47–58, see the inset of Figure 6a), which is held in place by the cis mixed αβ domains. The geometry of the lateral cavity is wedged and inclined, with a moderately polar surface composition. D155 is the only exposed charged side chain, while K49 and E129 form a stable salt bridge and are only partially solvent accessible, Figure 6b. Several surface charges are present in the lumen and are marked in Figure 6b with blue and red asterisks. The β-barrel is overall negatively charged with four acidic residues and two basic ones for each of the nine protomers. The cis vestibule has two acidic residues near the constriction. Other charged residues are located at the entrances of the cis and trans vestibules. Globally, the total pore charge is zero and the constriction has no charged residues exposed.

We performed a set of MD simulations at different applied voltages, in a 2 M KCl water solution. At equilibrium (Δ*V* = 0) the pore exhibits a net negative charge *q*_N_ in the constriction, Figure 6b,d. For Δ*V* > 0, *q*_N_ remains quite constant and the anion selectivity (Δ*J* < 0) shows a linear scaling with Δ*V*, Figure 6e. EOF is negative since the water flow follows the motion of the anions, Figure 6f. For small negative Δ*V*, the pore is still anion selective (*q*_N_ < 0 and Δ*J* < 0) and *v*_eo_ becomes positive since, again, the water flow follows the motion of the anions. This is the usual behavior of an electroosmotic flow where the charge accumulation in the pore is due to a wall potential independent of the Δ*V*. An inversion of both the accumulated charge *q*_N_ and selectivity is observed for large negative voltages, Δ*V* < −0.5 V, consistently with the geometrically induced selectivity switch mechanism. Gray dashed lines in Figure 6d–f report the predictions of the theoretical model. For completeness, the current–voltage curve is reported in Supplementary Figure S14. Although the pore geometry is quite far from the ideal model system of Figure 1 and asymmetries are present in the curves, the simplified model is still able to capture the order of magnitude of the EOF. As in the solid-state nanopore with an asymmetric electrolyte discussed in Figure 5, the data suggest that the presence of an equilibrium (intrinsic) net charge in the pore results in a sort of shift of the EOF curve with respect to the theoretical parabolic prediction. In the solid-state case of Figure 5, the pore is intrinsically cation selective (at low Δ*V*) and the selectivity inversion occurs at a positive Δ*V*. Accordingly, the maximum of EOF is shifted toward positive Δ*V*. Conversely, in CsgG, the pore is intrinsically anion selective (at low Δ*V*), so the selectivity inversion occurs at a negative Δ*V* and the EOF curve is shifted toward the left.

Further details on the charge distributions for Δ*V* = 0 are reported in Figure 6b. The map shows several charge accumulation spots due to the solvent-exposed charged residues in the two vestibules. Another relevant difference with respect to the ideal solid-state case is the charge distribution in the constriction at equilibrium (Δ*V* = 0), which shows a relative accumulation of positive (negative) ions on the trans (cis) side of the constriction. This peculiar distribution and the consequent intrinsic anion selectivity may reflect the complex shape of the constriction and the different hydropathy of the surface, composed of hydrophilic (Y51 and N55) and hydrophobic (F56) parts; see the inset in Figure 6a. Nevertheless, in agreement with our induced charge model, when an external Δ*V* is applied, ions accumulate in the lateral cavity of CsgG (altering also the charge distribution in the constriction), as shown in Figure 6c. This voltage-dependent behavior is better highlighted by Figure 6g, representing the difference of the maps at Δ*V* = 1 V and Δ*V* = −1 V. An alternative representation of the differential maps with respect to the equilibrium (0 V, Figure 6b) is reported in Supplementary Figure S14. For comparison, we also ran simulations for a neutralized pore. Charge accumulation spots in the pore vestibules are much less evident; nevertheless the charge distribution in the constriction is quite similar to the unmodified CgsG and, consequently, ion currents, selectivity, and EOF are, in essence, unchanged; see Supplementary Figure S15. In addition, in Supplementary Figure S16 we also reported an analysis that attempts to compare the induced charge EOF predicted by our geometrical model (that scales as Δ*V*^2^) and the expected linear EOF due to intrinsic anion selectivity at different voltages. This analysis indicates that for |Δ*V*| ≲ 0.3 V the dominant contribution is the intrinsic selectivity, while for |Δ*V*| ≳ 0.3 V, the induced charge mechanism dominates the EOF. MD data for negative Δ*V*, where selectivity inversion is observed, approximately supports this theoretical threshold. Although 0.3 V is larger than the typical Δ*V* employed in biopore experiments, we mention that polymeric membranes^56^ allowed biological nanopore experiments at Δ*V* ≈ 0.3–0.4 V. In addition, peculiar decoration of solid-state supports for membrane anchoring permitted reaching the same voltages for both lipid^57^ and diblock copolymer^58^ membranes.

## Conclusion

We presented a mechanism of geometrically induced selectivity that switches with the applied voltage polarity in uncharged cylindrical nanopores, giving rise to unidirectional electroosmotic flow. We derived an analytical model and we tested our predictions against molecular dynamics simulations. The phenomenon is robust under variation of the system geometry (*e*.*g*., cavity size, pore length) and is shown to be applicable in real-word settings, *i*.*e*., with asymmetric electrolytes and weakly charged pores. Our model provides a quantitatively accurate estimation of the electroosmotic velocity that can be used for nanopore system design. Unidirectional electroosmotic flow also occurs for a biological pore, the CsgG protein, whose shape resembles the cavity–nanopore ideal system but where, as usual for biopores, a complex surface charge pattern is present. A similar pore structure is also found in other secretion-related proteins of known structure, such as InvG^59^ and PilQ^60^ secretins, extending the possibility to use biomolecular scaffolds to achieve geometrically induced selectivity. Moreover, the surface patterning needed to elicit this effect is achievable by modern nanofabrication technology, such as electron beam decoration of graphene,^61^ focused ion beam,^62^ or electron beam lithography, reactive ion etching of TEM-drilled silicon nitride membranes.^43^ The mechanism we unraveled allows inducing a tunable ion selectivity even without altering the pore shape, surface charge, or chemistry, and, consequently, it may be useful for a more flexible design of selective membranes. The magnitude of the EOF associated with geometrically induced selectivity is comparable to other more common sources of EOF such as fixed surface charges^20,22,23,63,64^ and, by appropriate choice of settings, can even dominate them. Consequently, we expect that such a mechanism may find application in all the technologies where EOF is already used. One example is alternate current electroosmotic pumps,^35,65,66^ where different mechanisms have been exploited to induce a net EOF from a zero average oscillating potential in micro-^66^ and nanofluidic^35,65^ systems. In this respect, the average EOF intensity for a membrane constituted by conical nanopores^65^ is on the same order as the one we observed. Similarly, our mechanism may be employed in nanopore-based single-molecule sensing devices, where calibrating the competition/cooperation between electroosmosis and electrophoresis^20,21^ is crucial to control particle capture, especially for neutral or weakly charged molecules such as proteins and peptides.^22,23^ Since the EOF is induced without modification of the pore interior, in principle the geometric mechanism we propose to generate selectivity and electroosmotic flow may allow to separately and independently engineer the pore lumen to improve the sensing performance and the external cavity to control EOF.

## Methods

### General Molecular Dynamics Simulation Methods

All MD runs were carried out using NAMD,^67^ using a time step of Δ*t* = 2.0 fs and particle mesh Ewald^68^ method with a 1.0 Å spaced grid for long-range electrostatic interactions. A cutoff of 12 Å with a switching distance of 14 Å was set for the short-range nonbonded interactions. Periodic boundary conditions with a hexagonal prism cell are used unless otherwise stated. A Langevin thermostat was used for all the simulations. Nosé–Hoover Langevin piston pressure control was used for constant pressure simulations.^69^

### Solid-State Pore Setup

Our model system, represented in Figure 1d, is composed of a hexagonal solid membrane of thickness *L* with a cylindrical nanopore of radius *R*, surrounded by a coaxial cavity of width *w* and height *d* = *L* – *h*, at a distance *s* from the nanopore wall. The hexagon apothem *a*_h_ (see the top-view inset of the membrane in Figure 1d, green line) is *a*_h_ = 2.1(*R* + *s* + *w*). The membrane is composed of hexagonally packed uncharged atoms; see Supplementary Figure S4. For Figures 1–3, the membrane is immersed into a 2 M electrolyte solution, composed of a symmetrical polar fluid (see below) in which oppositely charged ions are dissolved. For Figure 5, the membrane is immersed in a 2 M KCl water solution, using standard CHARMM parameters for TIP3P water molecules and potassium (K^+^) and chloride (Cl^–^) ions. The *z*-dimension of each simulation cell is about *H*_*z*_ = 2*a*_h_ + *L*, with *L* being the height of the membrane, to ensure that the liquid height surrounding the pore entrance is greater than two times the pore diameter. The system is equilibrated with a constant pressure (flexible cell NPT) run at *P* = 1 atm and *T* = 250 K, keeping the *x*,*y* plane area fixed. The production runs are conducted at constant volume, temperature, and particle number (NVT ensemble), with a constant and homogeneous electric field **E** = (0, 0, *E*_*z*_) applied to charged atoms.

### Model Dipolar Fluid

The model fluid is composed of diatomic molecules, each formed by two atoms of mass *m* = 10 Da, of opposite charge *q*^+^ = 0.5*e* and *q*^–^ = −0.5*e*, covalently bound through a harmonic potential *U* = *k*_b_(*r* – *r*_0_)^2^ where *r* is the distance between the two atoms, *r*_0_ = 1 Å the equilibrium distance, and *k*_b_ = 450 kcal/(mol Å^2^) the spring constant; see Supplementary Figure S4. Intramolecular interactions are modeled *via* a standard Coulomb potential plus a Lennard-Jones (LJ) potential, with ϵ_LL_ = 0.1 kcal/mol and σ_LL_ = 2.68 Å. The above parameters were chosen to have volume, dipole moment, and mass similar to those of TIP3P water.^70^ The fluid exhibits a stable liquid phase in the temperature range 200 ≤ *T* ≤ 400 K, under a pressure of *P* = 1 atm; see the phase diagram in Supplementary Figure S5. At *T* = 250 K, the liquid density is ρ = 55.5 mol/L while the relative electric permittivity is ε_L_ = 83.2 ± 4.6 and dynamic viscosity η = 0.35 ± 0.02 mPa s. Relative permittivity ε_L_ was assessed by computing the dipole moment fluctuations in equilibrium NVT MD simulations;^71^ nonequilibrium estimations lead to similar results, Supplementary Figure S6. Viscosity η was estimated by applying a shear stress on the top of a liquid volume and measuring the slope of the resulting velocity profile (Couette flow), Supplementary Figure S7.

Nonbonded interactions between fluid and solid molecules were modeled using an LJ potential, with ϵ_SL_ = 0.8ϵ_LL_ and σ_SL_ = σ_LL_ (SL, solid–liquid), resulting in a hydrophilic pore. The wettability of the solid was assessed by evaluating the contact angle θ of a cylindrical drop of fluid onto the surface as a function of temperature and liquid–solid interaction potential ϵ_SL_;^72^ see Supplementary Figure S8. For the selected ϵ_SL_/ϵ_LL_ ratio, the contact angle is θ ≃ 60°.

The dissolved ions are composed of monovalent charged particles with charges *q*^±^ = ±1*e* and mass 40 Da. Nonbonded interactions of each ion with other atoms are described in Supplementary Figure S4. The ion diffusion coefficient for a 2 M solution at *P* = 1 atm and *T* = 250 K is *D* = 94.4 ± 0.7 Å^2^/ns, corresponding to an ion mobility of μ = 4.4 × 10^3^ Å^2^/(V ns); the diffusion coefficient *D* is estimated from the mean squared displacement (MSD); see Supplementary Figure S9.

### CsgG Pore Setup

The membrane–CsgG system was assembled using a protocol similar to the one reported in refs (73) and (74). The system was built starting from the CsgG X-ray crystal structure taken from the Protein Data Bank (PDB_ID: 4UV3(37) downloaded from the OPM database).^75^ The beta-barrel missing fragments (F144, F193 to L199) are modeled by using the SWISS-MODEL server.^76^ Other missing fragments (V258 to S262), located in the periphery of the cis side of the pore, were deemed to be not important for the ion and EOF transport and were not taken into account. The POPC lipid membrane, the water molecules, and the ions to neutralize the system were added using VMD (visual molecular dynamics).^77^ Salt concentration was set to 2 M KCl. The CHARMM36 force field^78^ was employed to model lipid, protein, and TIP3P water molecules.^70^ Nonbonded fix corrections were applied for ions.^79^ All covalent bonds with hydrogen were kept rigid, using SETTLE^80^ for water molecules and SHAKE/RATTLE^81^ for the rest of the system.

The energy of the system was first minimized for 10 000 steps using the conjugate gradient method. Then a pre-equilibration of 1 ns is performed to let the lipid tails melt and the electrolyte relax: the temperature was increased from 0 to 300 K in 100 ps, and then the Langevin thermostat with a damping coefficient of 1 ps^–1^ was applied to all non-hydrogen atoms; external forces were applied to the water molecules to avoid their penetration into the membrane, while the backbone of the protein and the lipid heads were constrained to their initial positions by means of harmonic springs, *k*_b_ = 1 kcal/(mol Å^2^); the Nose–Hoover Langevin method, with a period of 100 fs and decay of 50 fs, was used to keep a pressure of 1 atm, allowing the unit cell volume to fluctuate, by keeping the ratio between the *x* and *y* axes constant. A second equilibration run of 1.3 ns was performed to compact the membrane, letting the lipid heads remain unconstrained and reducing the spring constant on the protein backbone to *k*_b_ = 0.5 kcal/(mol Å^2^), until the three unit cell vectors reach a stationary value. The last equilibration step consisted of a 3 ns NPT run (as in the previous step, keeping the ratio between the *x* and *y* axes constant) where all the atoms were unconstrained and no external forces were applied to the water molecules. At the end of the equilibration procedure, the hexagonal periodic box has the following basis vectors: *v*_*x*_ = (179, 0, 0) Å, *v*_*y*_ = (89, 155, 0) Å, and *v*_*z*_ = (243, 0, 0) Å, for a total of 680 827 atoms.

### Current Measurements

The production runs were performed at constant volume, temperature, and particle number (NVT ensemble). The length of each simulation is indicated in the caption of the figures. For each case, a uniform and constant external electric field **E** = (0, 0, *E*_*z*_) was applied perpendicularly to the membrane. This protocol was shown to be equivalent to the application of a constant voltage Δ*V* = *E*_*z*_*L*_*z*_(82) (*E*_*z*_ > 0 for Δ*V* > 0, as indicated in Figure 1b). In the solid-state nanopores, the solid atoms are constrained to initial lattice positions with a harmonic spring, *k*_b_ = 100 kcal/(mol Å^2^), the solid membrane is thermostated, and coordinates are saved every Δ*t* = 50 ps. In the CsgG case, lipid head phosphorus atoms are harmonically constrained to the position of the last configuration of the equilibration phase, with *k*_b_ = 10 kcal/(mol Å^2^), and a thermostat is applied to the lipid and protein atoms (not hydrogens). Snapshots are saved every Δ*t* = 40 ps. The average current in the interval [*t*, *t* + Δ*t*] is estimated as^73,74,83^
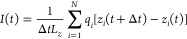
11where *q*_*i*_ and *z*_*i*_ are the charge and the *z*-coordinate of the *i*th atom, respectively. Ionic currents (either K^+^ and Cl^–^ or model ions) were computed by restricting the sum over the atoms of corresponding type.^73^ The mean current is obtained *via* a block average of *I*(*t*) (each block corresponding to 10 ns) after discarding a transient of 30 ns. The EOF is measured similarly, computing the summation over the fluid atoms and using the mass instead of the charge in eq 11. The results are then converted from mass flow rate to volumetric flow rate using the bulk liquid density.

### Charge Density, Velocity Fields, and Potential Maps

Using the VMD Volmap plug-in,^77^ we divided the system in cubic cells of size Δ*x* = Δ*y* = Δ*z* = 1 Å, and we calculated the average charge in each cell using the frames of the stationary state of the production run. A similar protocol is applied for the velocity profiles. In a given frame *f*, the velocity of the *i*th atom is computed as **v**_*i*_(*f*) = (**x**_*i*_(*f* + 1) – **x**_*i*_(*f* – 1))/(2Δ*t*), with **x**_*i*_(*f*) its position and Δ*t* the sampling interval. The average velocity in each cell is then calculated by averaging over the particles belonging to the cell and over time. The electric potential maps are computed by using the *pmepot* plug-in of VMD^73^ based on the particle-mesh Ewald method (PME). We then transformed the charge density and the velocity fields from the (*x*, *y*, *z*) Cartesian coordinate system to a cylindrical coordinate system (*r*, *z*, α) and performed a further averaging on α to get density and velocity fields in the (*r*, *z*) plane as the ones showed in Figure 1e,f and Figure 6b,c. Confidence intervals in Figure 1e were obtained using a block average with each block corresponding to 10 ns.

### Surface Charge Models

Functional models for the pH dependence of the surface charge σ_w_ for solid-state SiN nanopores, used in Figure 4, were taken from the experimental works of Lin *etal*.^44^ and Bandara *etal*.^45^ These models are used to fit experimental conductance data measured at different pH for different nanopore setups. In particular, for the black curve of Figure 4b we used the expression reported in eq 8 of ref (44) together with the fitted values reported in the Figure 3a of the same paper. For the red curve of our Figure 4b and all the curves of Figure 4c, we used the expression eq 3 of ref (45) using for each system the respective fitted parameters reported in the Supporting Information of the same work.

## Supporting Information Available

Details of the calculation of induced Debye layer capacitance for the cavity–nanopore system; PNP–NS model for EOF and comments on the model assumptions; characterization of our atomistic model for symmetric electrolyte solution in terms of phase diagram, relative electrical permittivity, wetting, ion mobility, and viscosity; ion currents as a function of the voltage for our model system and for the CsgG nanopore; electric potential for different cavity sizes; EOF prediction for a neutral silicon nitride nanopore of radius 20 nm; MD simulations of weakly charged nanopores; alternative maps of Figure 6g; fluxes and charge density maps for a neutral model of the CsgG nanopore; comparison between parabolic induced charge and linear fixed charge EOF; table reporting surface charges for solid-state nanopores (PDF)

## Notes

A prereview version of the manuscript has been published on the arXiv preprint server: Giovanni Di Muccio; Blasco Morozzo della Rocca; Mauro Chinappi, Geometrically Induced Selectivity and Unidirectional Electroosmosis in Uncharged Nanopores. 2021, 2104.03390. arXiv.org, https://arxiv.org/abs/2104.03390 (accessed April 15, 2022).
