# Supplementary material for: Geometrically Induced Selectivity and Unidirectional Electroosmosis in Uncharged Nanopores
Source: ACS Nano. 2022 May 19;16(6):8716–28. doi: 10.1021/acsnano.1c03017 (PMC9245180; doi:10.1021/acsnano.1c03017)
Supplement: Supplementary file 1 — nn1c03017_si_001.pdf [file nn1c03017_si_001.pdf]

Supplementary Information: Geometrically Induced Selectivity and Unidirectional Electroosmosis in Uncharged Nanopores

Giovanni Di Muccio[1], Blasco Morozzo della Rocca[2], Mauro Chinappi[1*]
[1] *Dipartimento di Ingegneria Industriale,*
Università di Roma Tor Vergata,
Via del Politecnico 1, 00133, Rome, Italy.
[2] *Dipartimento di Biologia,*
Università di Roma Tor Vergata,
Via della Ricerca Scientifica 1, 00133, Rome, Italy.

(Dated: May 8, 2022)

Contents:

Supplementary Note S1: Induced Debye layer capacitance for the cavity-nanopore. p. S-2

Supplementary Note S2: Comments on the PNP-NS model p. S-7

Supplementary Figure S1: Induced charge model and equivalent capacitance. p. S-4

Supplementary Figure S2: Electroosmotic velocity profile and Flow rate. p. S-8

Supplementary Figure S3: Role of prefactor on EOF and charge estimations. p. S-9

Supplementary Figure S4: Solid and dipolar fluid models. p. S-10

Supplementary Figure S5: Phase diagram for model dipolar fluid. p. S-11

Supplementary Figure S6: Dielectric constant for model dipolar fluid. p. S-12

Supplementary Figure S7: Viscosity of the model dipolar fluid in the liquid state. p. S-13

Supplementary Figure S8: Wettability of solid-state membrane by model dipolar fluid. p. S-14

Supplementary Figure S9: Ion diffusion coefficients and mobility for the model solution. p. S-15

Supplementary Figure S10: Ionic currents for model system and symmetric electrolyte. p. S-16

Supplementary Figure S11: Electric potential and Field lines for different cavity sizes p. S-17

Supplementary Figure S12: EOF predictions for a silicon nitride nanopore. p. S-18

Supplementary Figure S13: MD simulation of a weakly charged model pore p. S-19

Supplementary Figure S14: Net charge distribution and ionic currents for CsgG. p. S-20

Supplementary Figure S15: WT vs. Neutral Model of CsgG nanopore. p. S-21

Supplementary Figure S16: Comparison of Induced Charge and Intrinsic Selectivity of CsgG nanopore. p. S-22

Supplementary Table S1: Examples of surface charge and point of zero charge for some solid-state nanopores. p. S-23

Additional References: p. S-24

* mauro.chinappi@uniroma2.it

SUPPLEMENTARY NOTE S1: INDUCED DEBYE LAYER CAPACITANCE

Here we provide details on the derivation of the expression of the equivalent capacitance C_s between the lateral cavity and the pore lumen, Eq. (2) of the manuscript. We first derive the capacitance of a planar wall separating two reservoirs containing an electrolyte solution, then we extend the discussion for a cylinder and, finally, we calculate the equivalent capacitance for the cavity-nanopore system.

Planar membrane. The capacitance of a planar membrane is a classical problem, see, *e.g.* Läuger *et al.* [S1], here revised for reader convenience. Let us consider the system, represented in Supplementary Fig. S1a, composed of an infinite neutral solid membrane of relative permittivity ε_S and thickness h, separating two reservoirs of a perfectly symmetric 1:1 electrolyte solution with relative permittivity ε_L and bulk concentration c_0. A voltage ΔV is applied between the two reservoirs. Without loss of generality, we assumed that the left side (G) is grounded. The membrane is parallel to the Oxy plane, and $z = 0$ ($z = -h$) corresponds to the interface between the right (left) reservoir and the solid membrane. The problem is hence one dimensional and all the variables depend only on the z coordinate. For small surface potential $\zeta_w \ll k_BT/e$, the variation of the electric potential $\phi(z)$ into the electrolyte solution (A and G domains) is ruled by the Debye-Hückel approximation of the Poisson-Boltzmann equation [S2]

$$\frac{d^2\phi}{dz^2} = \frac{1}{\lambda_D^2}\phi \ , \quad \text{(electrolyte solution G)} \ , \tag{S1}$$

$$\frac{d^2\phi}{dz^2} = \frac{1}{\lambda_D^2}\left(\phi - \Delta V\right) \ , \quad \text{(electrolyte solution A)} \ , \tag{S2}$$

where

$$\lambda_D = \sqrt{\frac{\varepsilon_0\varepsilon_L k_B T}{2(\nu e)^2 c_0}}$$

is the Debye length, ε_0 the vacuum electrical permittivity, ν the valence of the ions (1 in our case), e the elementary charge, k_B the Boltzmann constant and T the temperature. Inside the membrane, instead, the Poisson equation reduces to

$$\frac{d^2\phi}{dz^2} = 0 \ , \quad \text{(membrane S)} \ . \tag{S3}$$

Equations (S1)-(S3) are solved with the following boundary conditions,

$$\phi(z) = 0 \quad \text{for} \quad z \to \infty \ , \tag{S4}$$

$$\phi(z) = \Delta V \quad \text{for} \quad z \to -\infty \ , \tag{S5}$$

$$\varepsilon_S \frac{d\phi_S}{dz}\bigg|_{z=0} = \varepsilon_L \frac{d\phi_G}{dz}\bigg|_{z=0} \ , \tag{S6}$$

$$\varepsilon_S \frac{d\phi_S}{dz}\bigg|_{z=-h} = \varepsilon_L \frac{d\phi_A}{dz}\bigg|_{z=-h} \ , \tag{S7}$$

where (S6) and (S7) impose the continuity of the normal component of the electrical displacement vector $\mathbf{D} = \varepsilon_0\varepsilon_{L/S}\mathbf{E}$ at the interfaces between the domains G and S, Eq. (S6), and A and S, Eq. (S7), respectively. The solution of the system (S1)-(S3) with the boundary conditions (S4)-(S7) is composed of two exponential branches in the liquid reservoirs and a linear branch in the solid membrane. In particular, we get the following solution

$$\phi(z) = \zeta_w \exp\left[-\frac{z}{\lambda_D}\right] \ , \quad \text{for}\, z \geq 0 \quad \text{(electrolyte solution G)}, \tag{S8}$$

$$\phi(z) = \Delta V - \zeta_w \exp\left[\frac{(z+h)}{\lambda_D}\right] \ , \quad \text{for}\, z \leq -h \quad \text{(electrolyte solution A)}, \tag{S9}$$

$$\phi(z) = \zeta_w - \left(\frac{\Delta V - 2\zeta_w}{h}\right) z \ , \quad \text{for} \ -h < z < 0 \quad \text{(membrane S)} \ . \tag{S10}$$

where

$$\zeta_w = \left(\frac{\varepsilon_S}{\varepsilon_L}\right)\left(\frac{\lambda_D}{h}\right)\left(\frac{\Delta V}{1+\frac{2\lambda_D}{h}\frac{\varepsilon_S}{\varepsilon_L}}\right) \tag{S11}$$

is the magnitude of the difference between the bulk potential and the surface potential. In essence, ζ_w is the magnitude of the induced surface potential.

From now on, since the system is symmetric with respect to the membrane (*e.g.* the two electrolyte solutions in the reservoirs G and A are identical), we will focus only on the ground reservoir G. Once $\phi(z)$ is known, the charge density in the liquid, $\rho_{el}(z)$, is obtained as

$$\rho_{el}(z) = -\frac{\varepsilon_0\varepsilon_L\zeta_w}{\lambda_D^2} \exp\left[-\frac{z}{\lambda_D}\right] \ , \quad \text{for}\, z \geq 0 \ , \tag{S12}$$

where, again, we are using the Debye-Hückel approximation. The region in the electrolyte solution domain where the charge accumulates under the action of the applied voltage ΔV is known as induced Debye layer (IDL, colored area in Supplementary Fig. S1 and manuscript Fig. 1c). Integrating Eq. (S12) in the liquid reservoir, the total charge q for unit of surface in the IDL is

$$q_{pla} = \int_0^\infty \rho_{el}(z)\, dz = -\frac{\varepsilon_0\varepsilon_L}{\lambda_D}\zeta_w = -\left(\frac{\varepsilon_0\varepsilon_S}{h}\right)\left(\frac{\Delta V}{1+\frac{2\lambda_D}{h}\frac{\varepsilon_S}{\varepsilon_L}}\right) \ . \tag{S13}$$

so that the capacitance per unit area of the membrane is

$$C_{pla} = \left|\frac{q_{pla}}{\Delta V}\right| = \frac{\varepsilon_0\varepsilon_S}{h}\left(1+\frac{2\lambda_D}{h}\frac{\varepsilon_S}{\varepsilon_L}\right)^{-1} \ . \tag{S14}$$

The quantity q_{pla} is also indicated as induced charge since its presence depends on the application of an external electric field. For a review on the various induce charge electrokinetic phenomena (often indicated as ICEK in the literature), we refer the reader to [S3] and reference therein. For $\lambda_D \ll h$, Eq. (S14) reduces to the formula for the geometric capacitance per unit area of a planar capacitor of height h,

$$C_{pla} = \frac{\varepsilon_0\varepsilon_S}{h} \ . \tag{S15}$$

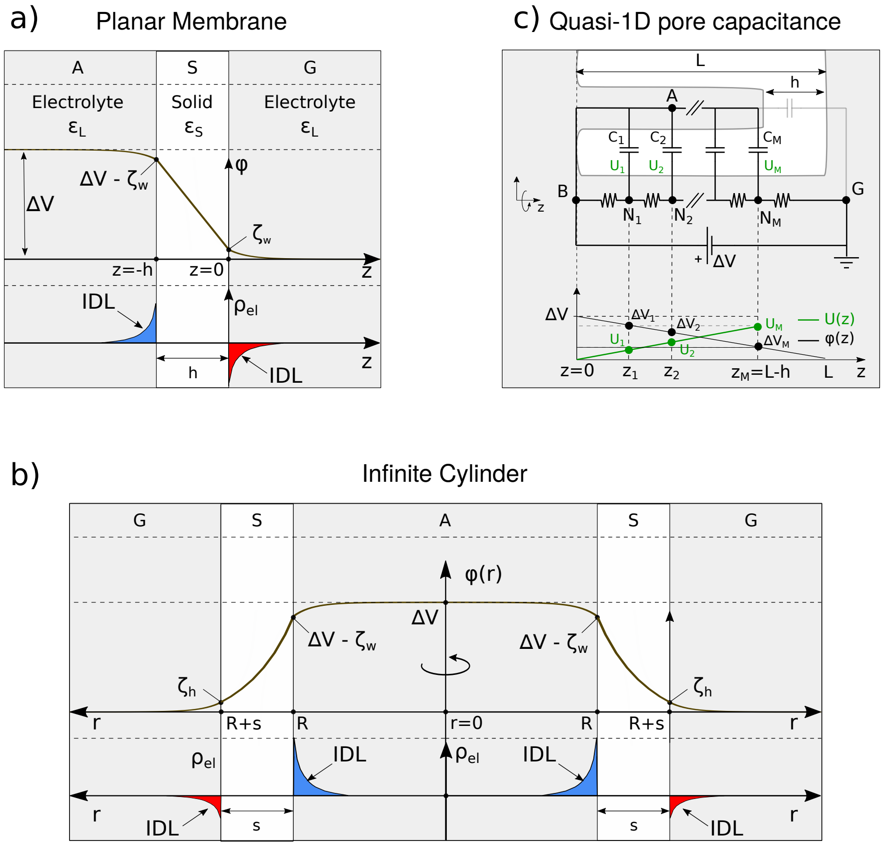

Supplementary Figure S1. Induced charge model and equivalent cavity-nanopore capacitance. a-b) Sketches of the electrical potential ϕ and net charge distributions ρ_{el} induced by a voltage drop ΔV across **a)** a planar membrane and **b)** an infinite cylinder, separating two electrolyte solutions (A and G). The continuity of the electrical displacement field at the solid-liquid interfaces drives the formation of Induced Debye Layer (IDL) of opposite charge, resulting in a capacitance. **c)** Circuit model employed for calculating the equivalent capacitance C_s between the pore lumen and the lateral cavity. The lower panel report the electric potential $\phi(z)$ (black line) that linearly decreases from ΔV (point B, $z = 0$) to 0 (point G, $z = L$) and the potential difference $U(z)$ (green line) between the lateral cavity and the pore lumen that linearly increases from 0 (point B, $z = 0$) to $\Delta V(L-h)/L$ (node N_m, $z = L-h$).

This is not surprising, indeed, for $\lambda_D \ll h$, the charge in the Debye layer accumulates in a very thin region close to the liquid-solid interface and $\zeta_w \ll \Delta V$, see Eq. (S11). In our system, λ_D and h are comparable, nevertheless, considering a 1M water solution $\varepsilon_L = 78$ of a 1:1 electrolyte at $T = 300$ K, ($\lambda_D = 0.304$ nm) and a $h = 1$ nm thick membrane with $\varepsilon_S = 1$, the second factor of Eq. (S14) is $\simeq 0.99$. So, again, Eq. (S15) is a very good approximation for the capacitance per unit surface. For this reason, Eq. (S15) is commonly used as starting point in the theoretical analysis of some induced charge electrokinetics phenomena, see, e.g. [S4], where it is employed to provide an estimation of the induced charge in a conical nanopore.

As a final comment, we note that, for the above mentioned systems (1M 1:1 electrolyte), for an applied voltage $\Delta V = 1$ V, Eq. (S11) predicts a value $\zeta_w \approx 3.4$ mV that is lower than the thermal voltage $k_B T/e = 25$ mV, implying that the Debye-Hückel approximation is valid even for relatively large voltages (compared to those often employed in nanopore experiments).

Infinite cylinder. Applying a similar approach, we derived an analogous result for a cylindrical electrolytic capacitance. Let us consider an infinite insulating cylindrical membrane of inner radius R and outer radius $R+s$ separating two electrolyte solutions (A and G), see Supplementary Fig. 1b. Outer domain is grounded (G) while the potential on the cylinder axis is ΔV. As in the planar case, in principle it is possible to solve the Debye-Hückel approximation of the Poisson-Boltzmann equation in the two domains A and G and the Poisson equation in the membrane domain S, Supplementary Fig. 1b. However, for $\lambda_D \ll R$ and $\lambda_D \ll h$, the IDL is thin compared to the cylindrical membrane size. So the capacitance for unit of length of the system can be approximated to

$$C_{cyl} = \frac{2\pi\varepsilon_0\varepsilon_S}{\ln\left(1+\frac{s}{R}\right)} , \tag{S16}$$

that, similarly to the planar case, is the geometrical capacitance for unit of length for a cylindrical capacitor with dielectric constant ε_S, inner radius R and outer radius $R+s$.

Equivalent cavity-nanopore cylindrical capacitance. For the estimation of the capacitance between the nanopore lumen and the cavity surrounding the entrance, we developed the quasi-1D model sketched in Supplementary Fig. S1c. The white domain in Supplementary Fig. S1c corresponds to a half-section of the solid membrane separating the two electrolyte reservoirs. The nanopore connects the two reservoirs and its axis lies on the z axis of the coordinate system. In our circuit model, the branch BG corresponds to the pore lumen and the point A to the lateral cavity. The point G is connected to the ground and a potential ΔV is applied at the point B (left pore entrance, $z = 0$). We assume that the potential inside the lateral cavity is uniform and equal to ΔV, *i.e.*, equal to the potential at the left pore entrance. Instead, the potential $\phi(z)$ along the nanopore linearly decays from ΔV to zero, when moving from left entrance ($z = 0$) to right entrance ($z = L$)

$$\phi(z) = \Delta V\left(1 - \frac{z}{L}\right) . \tag{S17}$$

The difference in the potential between the pore lumen and the cavity (point A) leads to an accumulation of charges inside the pore lumen that can be estimated as follows. Let us divide the branch BG into M segments so that $M-1$ intermediate nodes N_k exist, each one at potential $V_k = \phi(z_k)$, with $z_k = k\Delta z$ and $\Delta z = (L-h)/M$, with L the pore length and h the membrane thickness in the cavity, see Supplementary Fig. S1c. For each node N_k, we assume a cylindrical capacitor C_k of length Δz, subjected to a potential difference $U_k = \Delta V - V_k$. The charge q_N is then the sum of the charges accumulated in each C_k capacity,

$$q_N = \sum_{k=1}^{M} C_{cyl}\, U_k\, \Delta z , \tag{S18}$$

with $C_{cyl} = C_k/\Delta z$ the cylindrical capacitance per unit length.

In the limit $M \to \infty$, we have $z_k \to z$, $U_k = U(z)$ and the summation becomes the integral

$$q_N = \int_0^{L-h} C_{cyl}(z) U(z)\, dz . \tag{S19}$$

In general $C_{cyl}(z)$ depends on the radius of the pore, see Eq. (S16), and hence, for a generic axial-symmetric pore shape, it is a function of z. However, in our case the pore is cylindrical so C_{cyl} is constant along the pore and Eq. (S19) can be analytically worked out as

$$q_N = C_{cyl}\int_0^{L-h} dz\, U(z) = \frac{1}{2}\frac{(L-h)^2}{L} C_{cyl}\Delta V \tag{S20}$$

that, using Eq. (S16), reduces to

$$q_N = \frac{\pi\varepsilon_0\varepsilon_S}{L}\frac{(L-h)^2}{\ln\left(1+\frac{s}{R}\right)}\Delta V , \tag{S21}$$

and consequently the equivalent capacitance is

$$C_s = \frac{\pi\varepsilon_0\varepsilon_S}{L}\frac{(L-h)^2}{\ln\left(1+\frac{s}{R}\right)} . \tag{S22}$$

In the Eq. (2) of the manuscript we added a prefactor $(L - 4\lambda_D)/L$ to take into account the effect of pore entrances. Indeed, as shown in Fig. 1e of the manuscript, the deformation of the charge distribution at the pore entrance extends for around $2\lambda_D$ inside the pore. In our systems, for 2M ion concentration $\lambda_D \simeq 2$ Å, for long pores (*e.g.* the $L = 30$ Å pore discussed in Fig. 1) this additional factor corresponds to a correction of $\simeq 25\%$ on pore lumen charge q_N and electroosmotic flow rate Q_w.

SUPPLEMENTARY NOTE S2: COMMENTS ON THE PNP-NS MODEL

To achieve practical analytical solutions such as Eq. (4) and Eq. (8), we needed to introduce several hypotheses in the electrohydrodynamical model. Here, for clarity, we revise the overall continuum theoretical framework on which our work is based. This framework is quite standard, we report here the main concepts, focusing on some hypotheses we find important to discuss, referring the interested readers to other sources such as [S2, S5].

As a first step, we present the continuum model. In electrohydrodynamics of electrolyte solutions, continuum models are derived by the momentum, mass, energy and species balance equations plus the Poisson equation for electrostatics. We indicate as c_α the ion concentration, with $\alpha \in [1, N_s]$ where N_s is the number of ion species. The first hypothesis we introduce is the dilution, *i.e.*, we assume that the concentration of ions is so low that the properties of the solutions (*e.g.* viscosity η, relative permittivity ε_L, density ρ) are not affected by c_α. Moreover, we assume that the solution is incompressible, hence the pressure p does not affect ρ. Then, we also assume that the variation of temperature T, due for instance to viscous dissipation or to heat flux at system boundaries, is so small that it does not affect the solution properties (ρ, η, ε_L, etc.). This last hypothesis allows to decouple the energy equation from the others, *i.e.*, mass, species and momentum balance can be solved without considering the temperature distribution. Dilution has two other relevant implications: i) the terminal velocity of a single ion of the solution due to an electric field $\mathbf{E}$ is strictly proportional to the electric field with the proportionality constant not affected by ion concentration c_α. ii) the diffusion contribution to the particle flux can be modelled using Fick's law with diffusion coefficient D not depending on c_α (these two claims are linked by fluctuation-dissipation relation). If we use, as is common for simple fluids, the Newtonian model for viscous stresses, the final set of equations reads

$$\frac{\partial c_\alpha}{\partial t} + \mathbf{u} \cdot \boldsymbol{\nabla} c_\alpha = D\nabla^2 c_\alpha + c_\alpha \mu \nabla^2 \phi \tag{S23}$$

$$\frac{\partial \boldsymbol{u}}{\partial t} + \boldsymbol{u} \cdot \boldsymbol{\nabla} \boldsymbol{u} = \frac{1}{\rho}\left(\eta \nabla^2 \mathbf{u} - \nabla p - \rho_e \boldsymbol{\nabla}\phi\right) , \tag{S24}$$

$$\nabla \cdot \boldsymbol{u} = 0 , \tag{S25}$$

$$\nabla^2 \phi = -\frac{\rho_e}{\varepsilon_L} , \tag{S26}$$

where $\boldsymbol{u}$ and ϕ are the fluid velocity and the electrostatic potential and ρ_e is the electric charge density which can be written as a function of the ionic species concentration c_α by

$$\rho_e = \sum_{\alpha=1}^{N_s} c_\alpha \nu_\alpha e , \tag{S27}$$

where $\nu_\alpha e$ is the charge of species α, expressed in terms of the elementary charge e, so, *e.g.* $\nu_\alpha = +1$ for K^+ and $\nu_\alpha = -1$ for Cl^-. Eq. (S23)-(S26) are commonly indicated as Poisson-Nernst-Planck-Navier-Stokes equations (PNP-NS). At sufficiently small scales, typical of nanopores, the Reynolds number is small and it is safe to neglect nonlinear and time-dependent terms, *i.e.*, the left-hand side of Eq. (S24), leading to the Stokes equation. Moreover, PNP-NS is a mean-field model, and, hence, correlations between the ions are neglected and well as fluctuation of the number of ions in the system. The latter issue are particularly relevant in nanopore systems. In particular, the particle number fluctuations in an open system of N particles scale as $\sqrt{N}$. In a nanopore, the number of ions is, in general, very low, and, in principle, fluctuations cannot be disregarded. Nevertheless, including fluctuation would lead to theoretical approaches that are difficult to implement analytically. For a discussion on the potential impact of such an assumption in nanofluidic systems and on alternative theoretical approaches, we refer the reader to the two reviews [S2, S6] and references therein. For generic geometries, the system (S23)-(S26) needs to be solved numerically. However, a specific solution exists in the Debye-Hückel approximation for the cylindrical pores whose surface is at a wall potential ζ_w under the action of an external electrical field parallel to the pore axis. This solution can be expressed in terms of Bessel functions [S5, S7], but, for $\lambda_D \ll R$, in essence the velocity profile is quite similar to a plug flow whose magnitude in the center of the channel is given by the Helmholtz-Smoluchowski electroosmotic velocity

$$|v_{eo}| = \frac{\varepsilon_0 \varepsilon_L |\zeta_w|}{\eta} \frac{|\Delta V|}{L} , \tag{S28}$$

while the charge profile is zero everywhere, except for a thin region close to the wall. Eq. (S28) corresponds to Eq. (5) of the manuscript, the only difference being that in Eq. (5) we reported the sign of the velocity in agreement with the reference system we selected, Fig. 1a-b. As λ_D/R decreases, the charge accumulated in the channel converges to the product of pore surface $2\pi RL$ times the surface charge of a planar Debye layer $\varepsilon_0\varepsilon_L\zeta_w/\lambda_D$. Eq. (9) of the manuscript is identical to Eq. (S28) when the expression $\sigma_w = \varepsilon_0\varepsilon_L\zeta_w/\lambda_D$ is used for nanopore surface charge.

Hence, in the derivation of our model we have two different sets of hypotheses. On the one hand, since we rely on PNP-NS model, we implicitly assume all the above-cited hypotheses (continuum, dilution, incompressibility, mean-field). Although the reliability of these assumptions at the nanoscale is *a priori* questionable, several studies reported an unexpected capability of PNP-NS model to capture quantitatively the current through nanopores. For instance, in Bonome *et al.* [S8], an analytical electroosmotic model based on ideal EOF in a cylindrical channel was shown to be able to capture the order of magnitude of EOF measured from MD simulation in an αHL nanopore. Other remarkable examples of successful application of continuum models to nanopores are the recent study by Willems *et al.* [S9], where the EOF through a large biological pore is calculated using a PNP-NS model, and the work by Wilson *et al.* [S10] where a steric exclusion model based on Ohm law allowed to calculate current blockades for proteins confined into a solid-state nanopore, open pore currents for biological channels and blockade currents produced by DNA homopolymers in MspA, showing impressive agreement with all-atom MD data. In both these works, a crucial ingredient to improve the quantitative matches of the continuum model with experiments or all-atom MD was the additional calibration of the transport coefficient (*e.g.*, the dependence of ion mobility on the distance from the wall and on the ion concentration). However, the qualitative trends are expected to be well captured also without these more detailed models, see, *e.g.*, Supporting Information of Willems *et al.* [S9]. The second class of hypotheses, instead, enters after PNP-NS and concerns the above-mentioned limit for $\lambda_D \ll R$. As shown in Fig. S2, these hypotheses tend to overestimate the EOF.

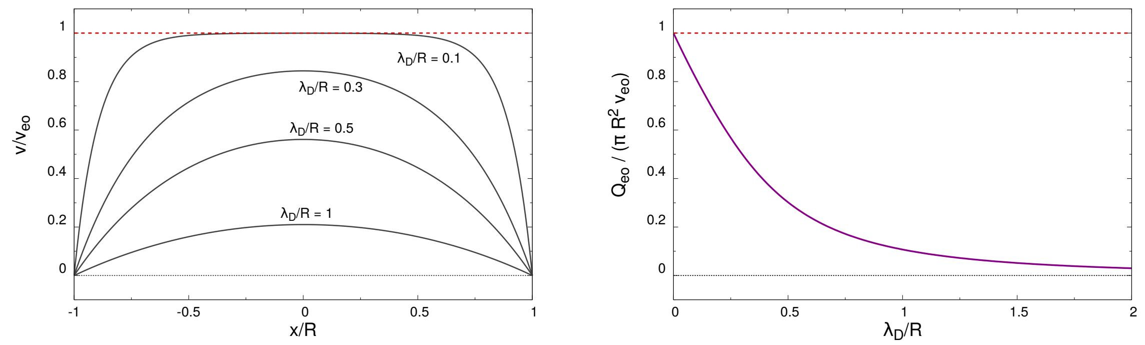

Supplementary Figure S2. Electroosmotic velocity profile and Flow rate. a) Electroosmotic velocity profile as a function of radial coordinate r for different values of λ_D/R. The curves refer to the classical analytical solution in a cylindrical pore of radius R with constant ζ_w at the wall and external electric field parallel to the pore axis. More specifically, $v = v_{eo}\left[1 - I_0(r/\lambda_D)/I_0(R/\lambda_D)\right]$ where I_0 is the modified Bessel function of order 0 [S5] and v_{eo} is the Helmholtz-Smoluchowski electroosmotic velocity [S5]. It is evident that for $\lambda_D/R \to 0$, the profile tends to a plug flow of velocity v_{eo}. **b)** Flow rate through a cylindrical pore as a function of λ_D/R, obtained integrating the velocity profiles $v(r)$ on the pore section. For $\lambda_D/R \to 0$, the mass flow rate coincides with the plug flow $Q_{eo} = \pi R^2 v_{eo}$, Eq. (5) of the manuscript.

The above-cited literature strongly suggested that our model would be able to describe the qualitative trends or to capture the order of magnitude of the selectivity and EOF. The fact that we also get a nice quantitative agreement with simulation data was not *a priori* expected. One possibility is that some fortuitous compensation happens. For instance, various overestimations of the EOF due to the assumption that $\lambda_D \ll R$ may be compensated by the prefactor $(L - 4\lambda_D)/L$ introduced to take into account the effect of pore entrances (see Supplementary Note S1). Nevertheless, also without the pore entrance prefactor, our model provides predictions that are quite close to the simulation data (the maximum difference is a factor 3 and occurs for very short pores where entrance effects are more relevant) see Supplementary Fig. S3, where

the same MD data of Fig. 3 of the manuscript are reported together with model prediction without pore entrance prefactor.

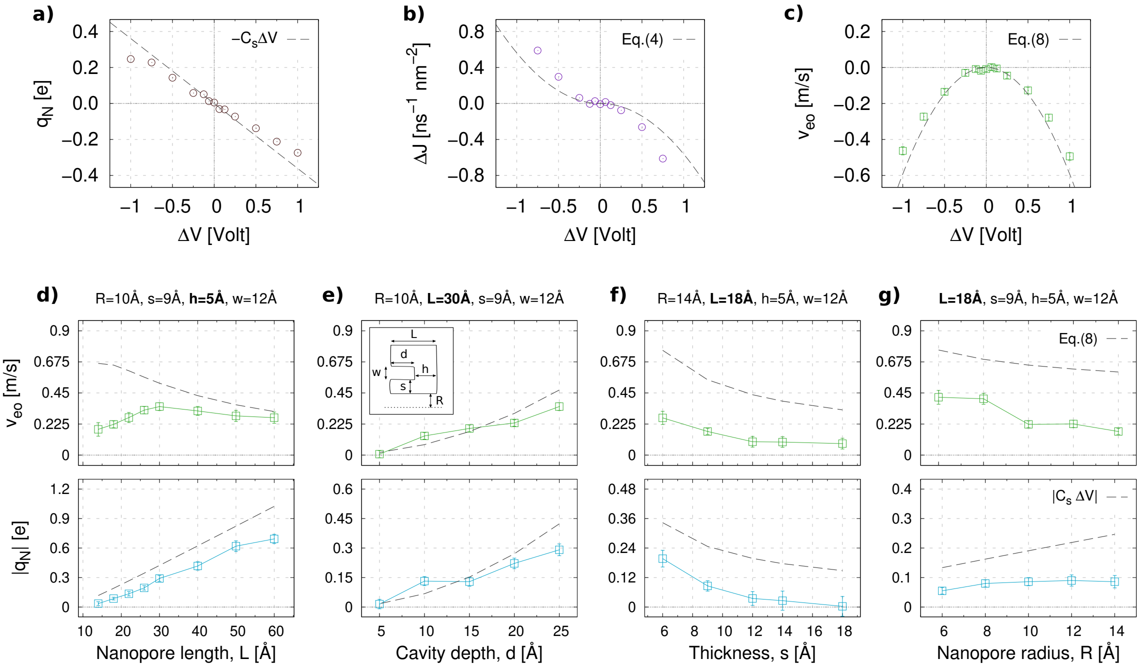

Supplementary Figure S3. Role of prefactor on EOF and charge estimations. The MD data reported are the same as in Fig. 3 of the manuscript, while the lines refer to the theoretical prediction obtained without the prefactor $(L - 4\lambda_D)/L$ introduced to consider the effect of pore entrance. In all cases, the model is able to capture the order of magnitude of EOF. The maximum difference is of about a factor 3 and occurs for short pores, see, *e.g.*, panels **f)** and **g)**, $L = 18$ Å and panel **a)** for $L < 25$ Å.

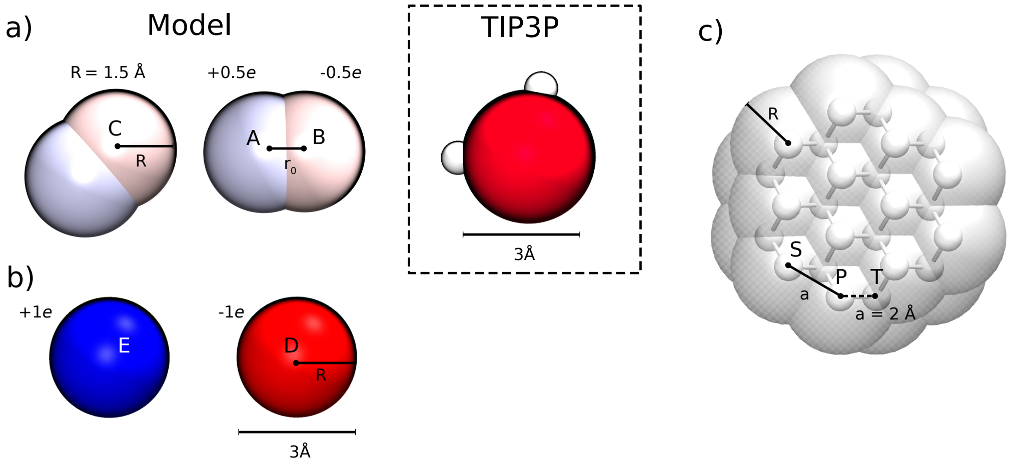

Supplementary Figure S4. Solid and dipolar fluid models. a) Diatomic particle forming our dipolar model fluid (the solvent). Each molecule is composed of two atoms covalently bound via a harmonic potential with spring constant $k_b = 450$ kcal/(mol Å^2) and equilibrium bond length $r_0 = 1$ Å. The two atoms carry opposite charges $q^+ = 0.5$e and $q^- = -0.5$e, giving an overall neutral molecule with a dipole intensity $|p| = 0.05$ e · nm. The mass of each atom is $m = 10$ Da. Non-bonded interactions, *e.g.* between atom A and atom C, are modeled using a standard Coulomb potential for the electrostatic forces and a Lennard-Jones (LJ) potential with parameters $\epsilon_{LL} = 0.1$ kcal/mol, $\sigma_{LL} = 2.68$ Å. On the right, a TIP3P water is drawn to scale. **b)** Ions are formed by atoms with charge $q^{\pm} = \pm 1$e and mass $m_I = 40$ Da. Non bonded interactions are again modeled as electrostatic plus LJ potentials. The LJ parameters for the ion-solvent interaction are the same as for solvent-solvent, while for ion-ion interaction we used $\sigma_I = 3.125$ Å. The slightly larger value σ_I compared to the solvent-solvent σ_{LL} was used to avoid the aggregation of ions. The resulting electrolyte solution corresponds to a fully dissociated binary symmetric salt in a symmetric liquid. **c)** Solid crystal forming the membrane. Top view of the hexagonal closest packed solid lattice, only two atomic layers are shown. Atoms are uncharged, with mass $m_S = 10$ Da and equilibrium distance of $a = 2$ Å. To keep the material solid, the atoms are harmonically bonded to three nearest neighbors belonging to the upper and lower planes (see *e.g.* atoms P and T) for a total of six bonds for each atom, with equilibrium distance $r_{0,s} = a$, and spring constant $k_b = 450$ kcal/(mol Å^2). LJ model is used for non-bonded interactions with $\sigma_{SS} = a$ and $\epsilon_{SS} = 1$ kcal/mol. In nanopore simulations the atoms are also constrained by a harmonic spring of $k_c = 100$ kcal/(mol Å^2). LJ parameters for solid-liquid interaction are $\epsilon_{SL} = 0.8\epsilon_{LL}$ and $\sigma_{SL} = \sigma$. The value of ϵ_{SL} can be modified to control the wettability, see Supplementary Fig. 8. Images are made using VMD [S11].

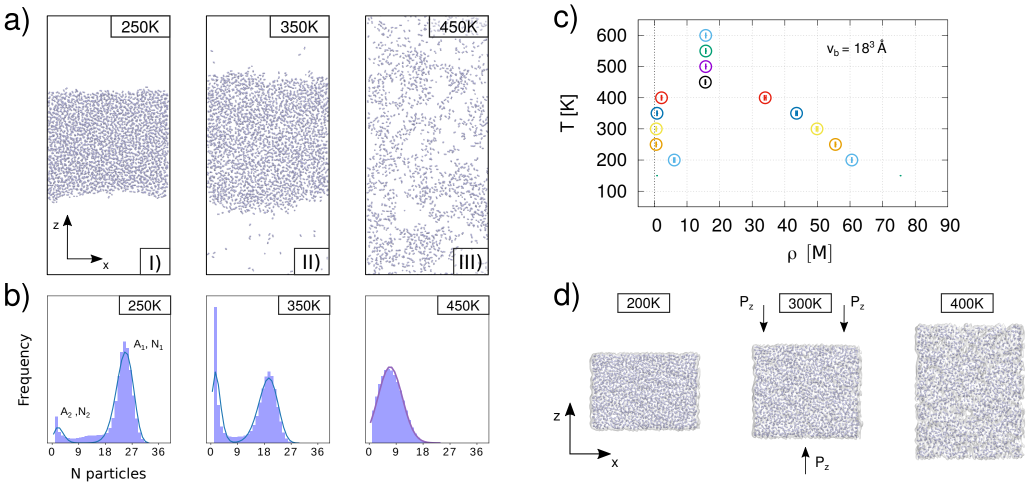

Supplementary Figure S5. Phase diagram for model dipolar fluid. NVT simulations of 20 000 dipolar molecules in a tripediodic box ($L_x = L_y = 90$ Å, $L_z = 270$ Å) were run at different temperatures T. A preliminary 200 ps pre-heating at 2500 K is followed by a 500 ps cooling to the final T. The pre-equilibration stage is used to bring the system away from local energy minima induced by the initial artificial arrangement of the molecules. Langevin thermostat with damping coefficient 0.05 ps^{-1} is used in all the runs. The equilibrated system is then sampled for 4 ns, saving the coordinates every 20 ps. **a)** Snapshots of the system at three representative T. **b)** Density distribution computed by dividing the simulation cell in cubic blocks of volume $v_b = 18^3$ Å^3 and counting the molecules in each block at every sampled frame. Solid lines refer to a bimodal Poisson fit (for bimodal distributions) or to a Gaussian fit (for unimodal distributions). **c)** Density-temperature state diagram. For each temperature, circles correspond to the peaks of the fitted distribution with error bars being the corresponding fit errors. Unimodal distributions are found for $T > 400$ K indicating that the critical temperature T_c is between $400 < T_c < 450$ K. Number density is reported in mol/L, for $T = 250$ K we get $\rho \simeq 55.5$ mol/L, a value very similar to water number density. **d)** Final frame of 12 ns NPT runs ($P = 1$ bar) at different temperatures, showing that our fluid is in a liquid state in the interval $200 \leq T \leq 400$ K. Panels **a** and **d** were made using VMD [S11]. Statistical analyses of density distributions were done using Python modules Scipy [S12] and Matplotlib [S13].

a)

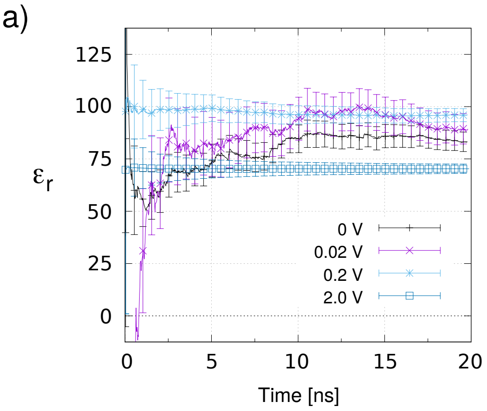

b)

ΔV	ε_r	err
0	83.2	4.6
0.02	89.3	7.2
0.2	96.1	3.1
2.0	70.3	2.2

Supplementary Figure S6. Dielectric constant for model dipolar fluid. a) The plot represents the MD relative permittivity estimation, using two different methods, as a function of the sampling time, for four different applied voltages ΔV. The simulation set-up is a rectangular box of size $L_x = L_y = 80$ Å, $L_z = 140$ Å. After an NPT equilibration ($T = 250$ K, $P = 1$ atm), a 25 ns NVT production run is started and frames are sampled every 20 ps. The first 5 ns were discarded. The first permittivity estimation method is a non-equilibrium approach where a constant electric field $\mathbf{E} = (0, 0, E_z)$, corresponding to a potential $\Delta V = -L_z E_z$ is applied to the system. Following [S14, S15], the relative permittivity ε_r is computed as

$$\varepsilon_r = 1 + \frac{\langle M_z \rangle}{\varepsilon_0 E_z \Gamma} ,$$

with ε_0 the vacuum permittivity, with M_z total dipole moment in the z-direction and $\Gamma = L_x L_y L_z$ the volume of the system. In the second method (black points) no electrical field is applied to the system, and we computed the dielectric constant as [S15]

$$\varepsilon_r = 1 + \frac{\langle M_z^2 \rangle}{\varepsilon_0 k_B T \Gamma} ,$$

with k_B the Boltzmann constant and T the temperature. It is apparent from the figure that the estimation of ε_r reached a plateau for all the simulated systems. Each data point is calculated as the average over the previous frames. The error bars represent the standard errors of the respective estimator, computed over the previous frames. The final estimated values, after 20 ns, are reported in panel **b)**. VMD [S11] is used to calculate M_z and M_z^2 at each frame.

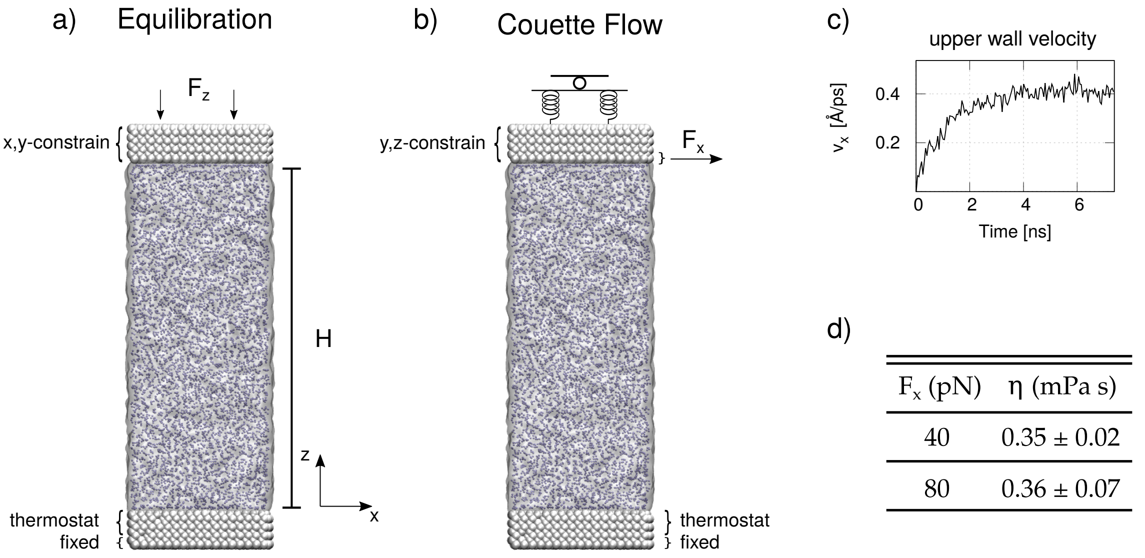

F_x (pN)	η (mPa s)
40	0.35 ± 0.02
80	0.36 ± 0.07

Supplementary Figure S7. Viscosity of the model dipolar fluid in the liquid state. The system is composed by 11 269 liquid molecules, confined between two flat solid slabs, each one composed of 6 936 atoms. The bottom layer of the lower wall is fixed, while atoms from the other layers are thermostated at $T = 250$ K (Langevin, damping 5 ps^{-1}). During the equilibration, panel **a)**, the atoms of the top layers are constrained on the x-y plane, while a force F_z, corresponding to a pressure of 1 atm, is applied along the z-axis to the topmost layer, until the liquid reaches a stable height $H = 134.69 \pm 0.02$ Å (density $\rho = 55.5$ mol/L). The production run is performed by applying a constant force F_x to the top solid layer in contact with the liquid, panel **b)**. During the production run, the atoms of the top layers are harmonically constrained on their $y - z$ positions, letting them free to move along the x direction. The top solid slab reaches a steady-state velocity v_x after about 6 ns, panel **c)**. After, a 2 ns sampling is performed to compute the velocity profile $v_x(z)$ of the liquid, saving the coordinates every 50 ps. For each frame, the velocity profile $v_x(z)$ is computed by dividing the volume height H into slabs of thickness $\Delta z = 1$ Å, using the following procedure: i) the atoms inside the slab at the frame f are selected; ii) the x-velocity of each atom k is computed as $v_k = (x_k(f+1) - x_k(f-1))/(2\Delta t)$, with $x_k(f \pm 1)$ x-position of the atom k at the frame $f \pm 1$, and Δt sampling interval; iii) the average velocity in the slab is computed as the average of the velocity of the atoms in the slab. By averaging over all frames after the transient, we get a linear profile $v_x(z)$ whose slope γ is related to the viscosity of the liquid $\eta = F_x/A\gamma$, with A surface area where the shear forcing F_x is applied. **d)** Values of viscosity η estimated at two different F_x.

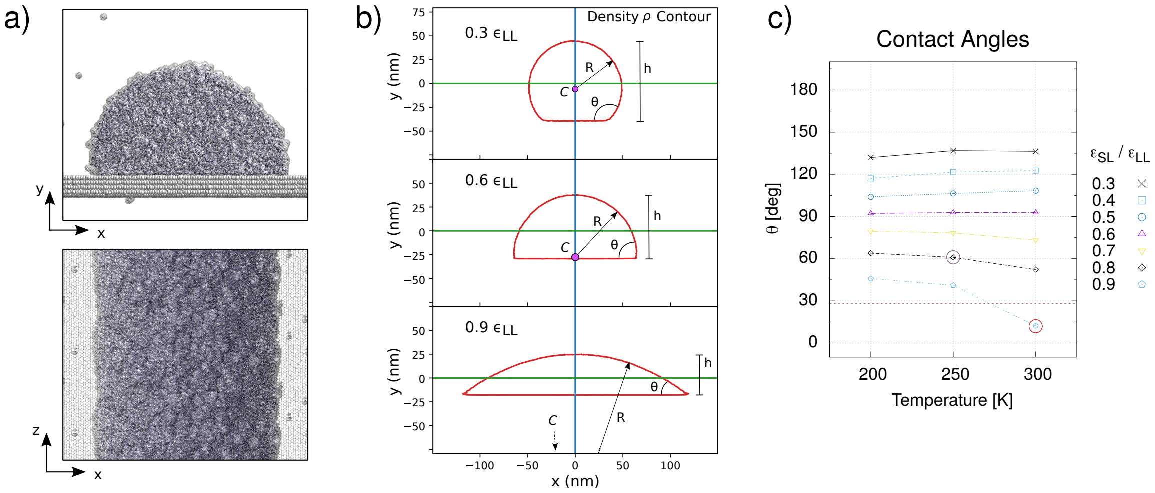

Supplementary Figure S8. Wettability of solid-state membrane by model dipolar fluid. a) System set-up for cylindrical droplet protocol. A liquid drop formed by 15 000 fluid molecules is in contact with a solid slab ($L_x = 200$ Å, $L_y = 60$ Å, 48 000 uncharged atoms, only the central portion of the system, where the drop is placed, is reported). Each solid atom is weakly constrained to its lattice position (harmonic spring constant $k_b = 100$ kcal/(mol Å^2). Wetting can be tuned with the liquid-solid LJ parameter ϵ_{SL}. The solid is eight atomic layers thick so that the slab z-height is greater than the LJ cutoff radius ($r_c = 12$ Å). Tri-periodic boundary conditions have been applied. To estimate the contact angle θ, NVT simulations are performed (2 ns equilibration, 4 ns production). During the production runs, a thermostat is applied only to the solid membrane. **b)** A procedure similar to one reported in [S16] is used to calculate θ. In brief, we first calculated a 3D density map of the liquid, discretizing the system in cells of $1 \times 1 \times 1$ Å^3 and averaging over time, at each step the droplet is centered on its center of mass to compensate for any collective movements of the fluid parallel to the solid surface. After, we computed a 2D density map by averaging over the z-planes. Droplet boundary is defined as the isodensity contour lines corresponding to the half-mode of the 2D density distribution (red contour), and θ is calculated as $\theta = \arccos(1 - h/R)$ with h and R the nominal height and the radius of the droplet. **c)** Contact angle θ as a function of the temperature T for different liquid-solid interaction ϵ_{LS}. The red circled point under the dashed line indicates that the liquid uniformly wets the entire surface. For our nanopore simulations, we used a $\epsilon_{SL} = 0.8\epsilon_{LL}$, corresponding to a hydrophilic surface with $\theta \simeq 60^\circ$, conditions indicated by the grey circled point.

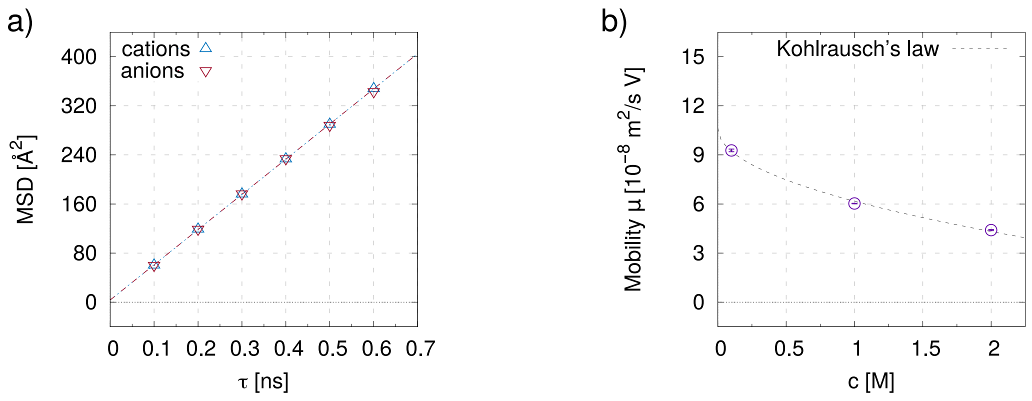

Supplementary Figure S9. Ion diffusion coefficients and mobility for the model symmetric electrolyte solution. a) Mean squared displacement (MSD) as a function of simulation time, for a 2M triperiodic system (313 292 total atoms, 11 280 ions). After an NPT equilibration ($T = 250$ K, $P = 1$ atm), a 4 ns NVT production run is performed, sampling particle positions every 100 ps. The first nanosecond is discarded and then the MSD is computed over the remaining frames by using VMD scripts [S11]. The slope of the curve is related to the diffusion coefficient D of the ion in the electrolyte solution by the relation $\mathrm{MSD}(\tau) = 6D\tau$ [S17], resulting in the fitted values $D_+ = 95.6 \pm 0.21$ Å^2/ns and $D_- = 94.4 \pm 0.73$ Å^2/ns. From the diffusion coefficient, the ion mobility is estimated via the Einstein relation $\mu_\pm = q_\pm D_\pm/(k_B T)$. Results at different concentrations are shown in **b)**. As predicted by Kohlrausch law [S18], $\mu(c) = \mu_0 - K\sqrt{c}$, the ionic mobility decreases with increasing ion concentration, with μ_0 the mobility at infinite dilution, and K an empirical constant.

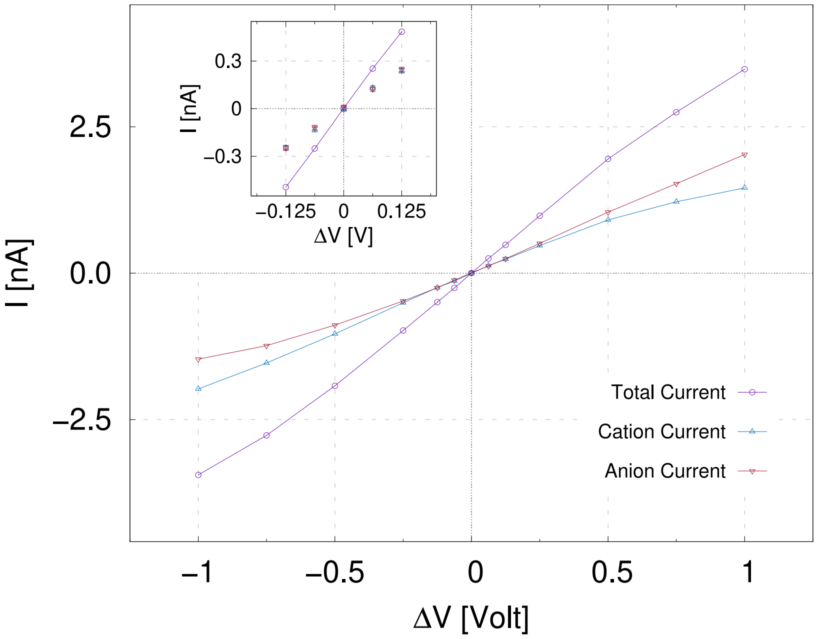

Supplementary Figure S10. Ionic currents for the model system shown in Fig. 1d-f and Fig. 3a-c of the manuscript. Currents are computed with the protocol described in the methods, averaging over 800 ns MD trajectories, for a total of 16 000 frames. The first 30 ns are discarded. Errors are smaller than the point sizes and are calculated using a block average protocol with a block length of 10 ns. It is apparent that for low voltages, $|\Delta V| \leq 0.125$V, the anion and cation currents are equal (see the inset). This is expected since, at low ΔV, the external voltage does not alter the equilibrium ionic distributions. Since the pore surface is neutral and the salt is symmetric, the distributions of anions and cations are identical at equilibrium. Moreover, in our model anion and cations have the same mobility, consequently the anion and cation currents have the same value.

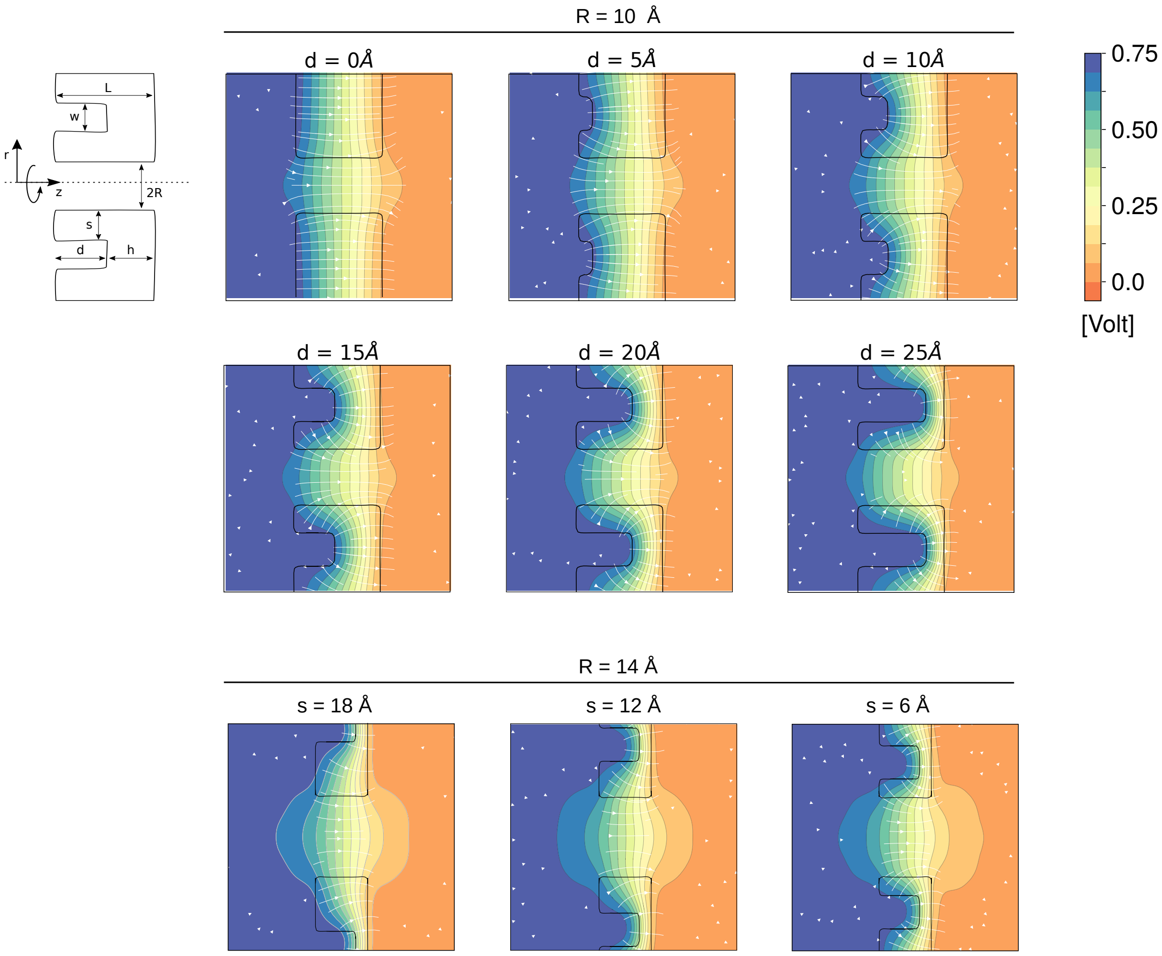

Supplementary Figure S11. Electric Potential and Field lines for different cavity sizes. The white arrowed lines represent the electric field $\mathbf{E}(r, z) = -\nabla V$. We filtered out the lines where $|\mathbf{E}(r, z)| < 15\%$ of the maximum intensity. The potential map is averaged over 800 ns MD trajectory (16 000 frames), at $\Delta V = +0.75\,\mathrm{V}$ transmembrane applied bias, in the presence of the model symmetric electrolyte. For the top group, the other geometric parameters are those of Fig. 3a-c of the manuscript, namely, $R = 10$Å, $L = 30$Å, $s = 9$Å, $w = 12$Å. For the bottom group the geometric parameters are those of Fig. 3f, namely $R = 14$Å, $L = 18$Å, $h = 5$Å, $w = 12$Å. The intensity of the electric field is larger in the solid region close to the cavity (where the membrane thickness is small) accordingly with the model already reported in Supplementary Note S1. The flux of the radial component of the electric field at the pore wall increases as the cavity depth d increases and as the coaxial separating membrane thickness s decreases (both for fixed L).

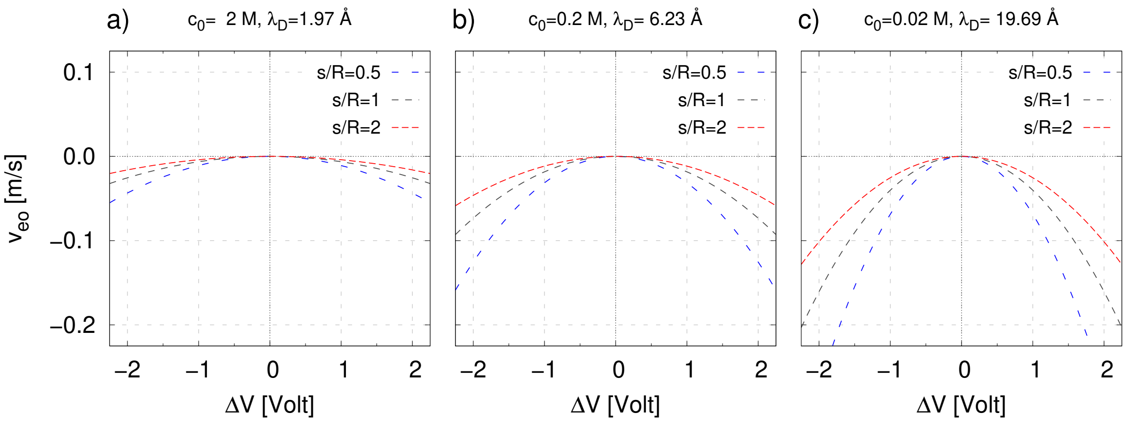

Supplementary Figure S12. EOF predictions for a neutral silicon nitride nanopore. We considered a pore of radius $R = 20$ nm and length $L = 20$ nm. The lateral cavity has a depth $d = 10$ nm and it is placed at a distance $s = 40$nm (red line, $s/R = 2$), $s = 20$ nm (grey line, $s/R = 1$) and $s = 10$ nm (blue line, $s/R = 0.5$). The relative electrical permittivity for solid and liquid are $\varepsilon_S = 7.5$ (silicon nitride) and $\varepsilon_L = 80$ (water). Panels represent different electrolyte concentration: **a)** 2 M, **b)** 0.2 M and **c)** 0.02 M. The corresponding Debye lengths λ_D are also reported on the top of each panel. The parabolic unidirectional EOF is obtained using Eq. (8) of the manuscript. Even for the lowest concentration (0.02 M), a total of $\simeq 600$ ions (300 for each species) are present inside the nanopore, *i.e.*, a number high enough to expect that a PNP-NS model provides a good estimation of the flows.

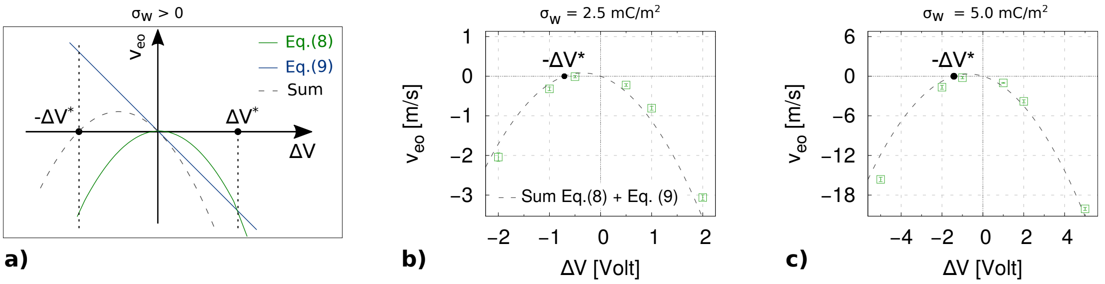

Supplementary Figure S13. MD simulation of a weakly charged model pore and analytical estimates. **a**) Sketch of the superposition of the two contributions of geometrically induced EOF, Eq. (8) of the manuscript (green parabola), and of fixed charge EOF, Eq. (9) (blue line), as a function of applied voltage ΔV. The resulting sum is shown as a dashed curve. Neglecting the trivial case at null voltage, there are two voltages in which the effects are equal in magnitude. For positive σ_w at ΔV = -ΔV^* the two contributions cancel out, yielding $v_{eo} = 0$. **b**) and **c**) MD simulation of a nanopore system similar to the one shown Fig. 1d of the manuscript, with geometrical parameters $R = 10$Å, $L = 30$Å, $h = 5$Å, $s = 9$Å, $w = 12$Å, modified by adding a charge density $\sigma_w = 2.5$ mC/m^2 (panel **b**) or $\sigma_w = 5.0$ mC/m^2 (panel **c**). The pore is immersed in 2M of our symmetric electrolyte and non-equilibrium simulations are performed as for the system of Fig. 1d-f of the manuscript, see Methods. The green squares represent MD data points. In several cases, error bars are much smaller than the data points. The dashed curve is an estimate of total EO velocity, calculated as a sum of the contribution due to fixed surface charge and the geometrically induced flow, by using the theoretical estimations reported in Eq. (8) and Eq. (9) of the manuscript, respectively. The threshold voltages $-\Delta V^*$, as estimated by Eq. (10) of the manuscript, are represented on the curves by a filled circle and labeled accordingly. The good agreement between the MD data and the theoretical predictions indicates that the theory, although derived under strong assumptions, provides a good description of the competition between fixed and induced charge effect, at least in the case of weakly charged pores.

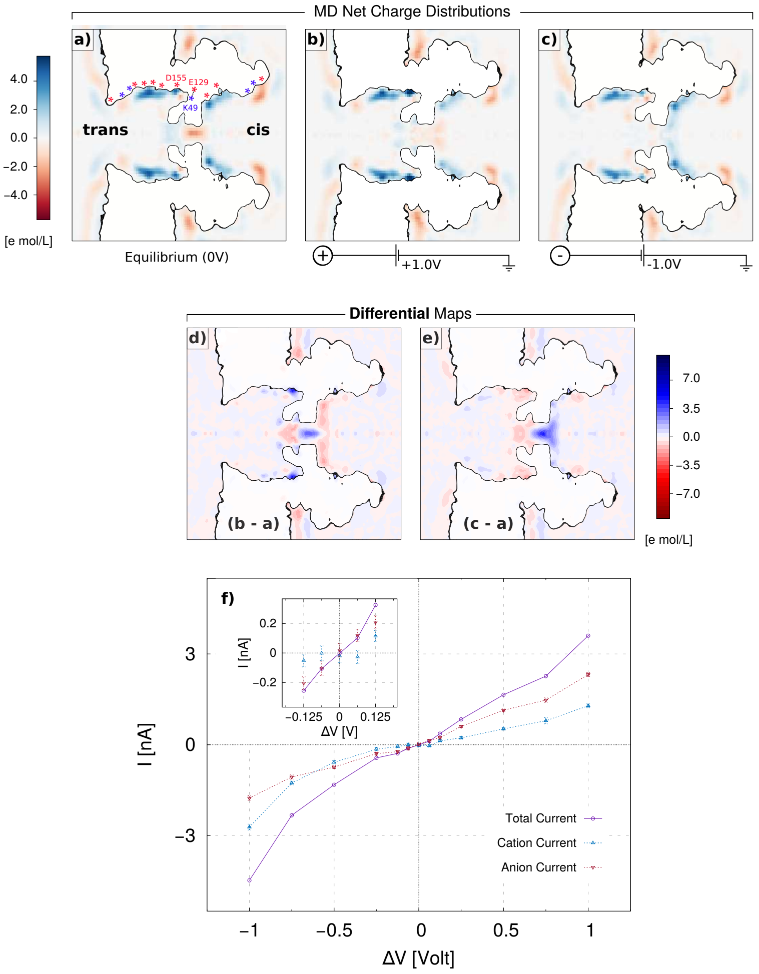

Supplementary Figure S14. Net charge distribution and ionic currents for the CsgG nanopore, Fig. 6 of the main text, computed from MD simulations in 2M KCl water solution. The first three panels (**a-c**) contain the maps already reported in Fig. 6**b-c**. Panels **d-e**, instead, represent the differential maps between the non-equilibrium systems ($\Delta V = \pm 1\,\mathrm{V}$, panels **b** and **c**) and the equilibrium one ($\Delta V = 0\,\mathrm{V}$). The black line delimiting the pore and the membrane is the water density contour level $\rho = 0.5\rho_{bulk}$, with ρ_{bulk} the bulk water density. Maps are obtained from 280 ns MD production runs. All the trajectories are sampled each 20 ps, and analyzed discarding the first 10 ns. The applied ΔV results in a strong reduction of the dipole in the pore constriction observed at $\Delta V = 0$, for this reason, the differential maps (**c - a**) and (**b - a**) present a dipole in the constriction. In the differential maps, it is also apparent that the cavity changes its charge when reverting the voltage. In particular, at $\Delta V = 1\mathrm{V}$ differential maps (**d**) show a positive variation of the cavity charge while the opposite occurs at $\Delta V = -1\mathrm{V}$ (**e**). Panel **f** reports the total electric current and single ionic currents. Currents and maps are computed with the protocols described in the methods, averaging over 280 ns MD production trajectories, for a total of 14 000 frames. All the trajectories are sampled each 20 ps, and the first 10 ns are discarded. Errors are calculated using a block average protocol with a block length of 10 ns.

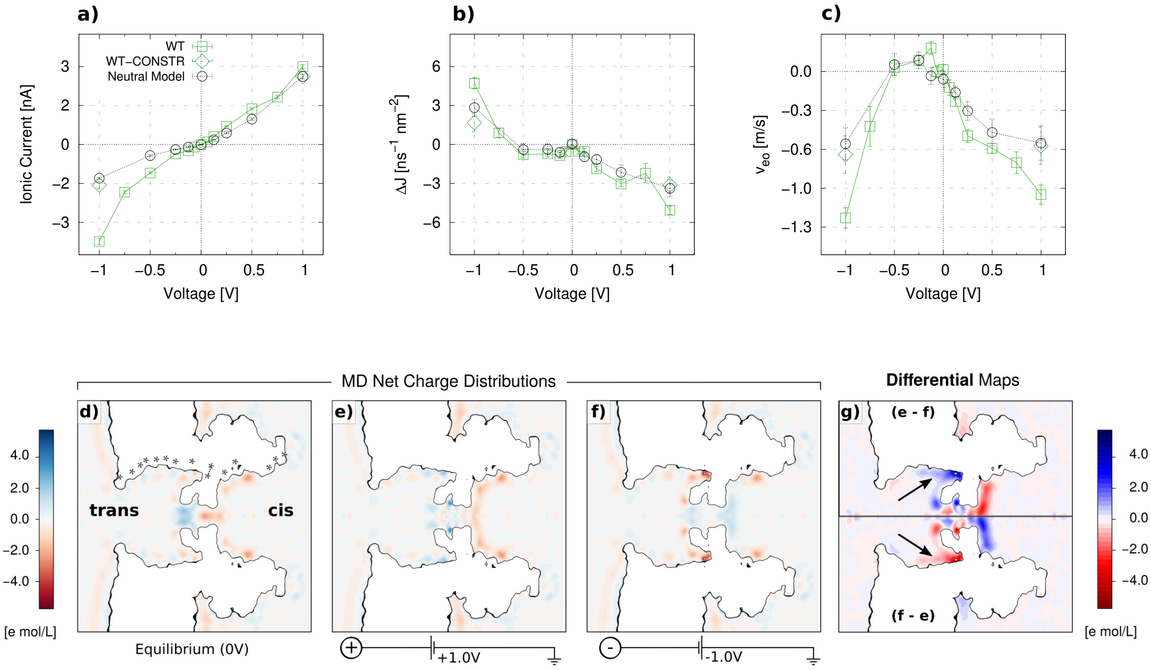

Supplementary Figure S15. Comparison between the CsgG nanopore, Fig. 6 of the manuscript, and a Neutral Model of the same pore. The Neutral Model is obtained by neutralizing the net charges of the acidic and basic residues, using standard neutral CHARMM patches of the charged residues. MD simulations are conducted in 2M KCl water solution, as for Fig. 6. The general trends for **a)** ionic currents, **b)** selectivity and **c)** electroosmotic velocity are similar to the Wild Type CsgG nanopore. The quantitative differences in the fluxes at high voltages are due to slight structural changes in the constriction. To prove this, we repeated the simulation of the wild type CsgG by keeping the structure constrained upon the Neutral Model. The results, green diamond points (WT-CONSTR) at $\Delta V = \pm 1.0V$, overlap with Neutral Models. Currents are computed with the protocol described in the methods. Each point is obtained by averaging over 240 ns MD trajectory, for a total of 120 000 frames. The first 10 ns are discarded. Errors are calculated using a block average protocol with a block length of 10 ns. Panels **d-g** report the charge density map for the Neutral Model. Charge distribution at the walls of cis and trans vestibule differs from the Wild Type (Fig. 6b-c of the manuscript). This is expected since the exposed residues that were originally charged in the Wild Type are now neutral. Instead, the charge distribution in the constriction is only slightly altered with respect to the Wild Type.

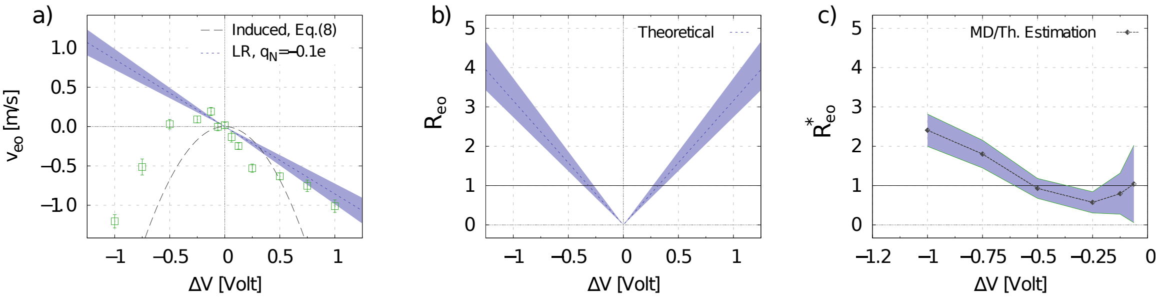

Supplementary Figure S16. Comparison between Induced Charge EOF and Intrinsic Selectivity EOF for the CsgG nanopore. To estimate the relative contribution of our geometrically induced mechanism on observed EOF we applied a simple additive model where the v_{eo} is interpreted as the sum of a linear response (LR) contribution due to intrinsic selectivity and the geometrically induced selectivity contribution. **a)** Electroosmotic average velocity v_{eo} as a function of the applied voltage ΔV. Green squared points and dashed grey line are the MD data and theoretical prediction of the geometrically induced charge model as reported in Fig. 6. The LR linear curve is obtained from Eqs. (5-6) of the manuscript, considering a fixed $q_N = -0.10 \pm 0.015$e inside the pore lumen. **b)** Ratio R_{eo} between the theoretical induced charge contribution $v_{eo,ICEO}$ (dashed parabola in panel **a**) and the intrinsic linear $v_{eo,LR}$ contribution to EOF (dotted line in panel **a**),

$$R_{eo} = \frac{|v_{eo,ICEO}|}{|v_{eo,LR}|} \, .$$

c) Ratio R^*_{eo} between the estimated induced charge contribution $v^*_{eo,ICEO}$ and $v_{eo,LR}$, where $v^*_{eo,ICEO}$ is computed at each ΔV as the difference between the MD measured EOF and the LR value, $v^*_{eo,ICEO} = v_{eo,MD} - v_{eo,LR}$. This expression, as well as the overall analysis discussed in this supplementary figure, implicitly assumes a superposition of effects, *i.e.* the total EOF can be decomposed as the sum of a linear contribution due to intrinsic selectivity and a quadratic contribution due to induced charge. This approach is similar to the one used in the section "Application to weakly-charged solid-state nanopores" of the manuscript and also discussed in Fig. S13, although in the case of CsgG the selectivity is not due to a net fixed charge present at the pore wall. This hypothesis is quite strong and, in general, unjustified. Consequently, these arguments can be used to get preliminary approximate voltage ranges where the intrinsic selectivity or the induced charge mechanism dominate the EOF, while simulations are needed for a reliable non-equilibrium description of the transport.

Paper	Material	Notes on surface charge σ_w and point of zero charge
Lin *et al.* [S19]	Silicon Nitride	From $\sigma_w = 0.0027$ C/m^2 (pH = 1.2) to $\sigma_w = -0.2$ C/m^2 (pH = 11). Point of zero charge at pH = 3.3 − 4.1 depending on the salt concentration. The paper reports an analytical model fitted on experimental data for the dependence of σ_w on the pH. This relation is used in Fig.4b of the manuscript.
Hoogerheide *et al.* [S20]	Silicon Nitride	From $\sigma_w = -0.04$C/m^2 (pH = 2) to $\sigma_w = 0.02$C/m^2 (pH = 8). Point of zero charge at pH $\simeq$ 4.1 measured at 0.11 M KCl.
Bandara *et al.* [S21]	Functionalized Silicon Nitride	The manuscript reports expressions for σ_w as a function of the pH. Using these expressions and the values reported in the Supporting Information of that manuscript, it is possible to calculate σ_w. Very small surface charge density is achieved for a wide range of pH, for instance, $\lvert\sigma_w\rvert < 0.001$ C/m^2 for pH < 7.5 for -OH functionalization and $\lvert\sigma_w\rvert < 0.003$ C/m^2 for pH > 7.5 for -NH_2 functionalization.
Kosmulski [S22]	HfO_2	From $\sigma_w = -0.1$C/m^2 to $\sigma_w = 0.1$C/m^2 depending on pH. Point of zero charge at pH $\simeq$ 7.5. This material was used for nanopore experiments in [S23].
Xie *et al.* [S24]	PET coated with CTAB	From $\sigma_w = -0.01$C/m^2 to $\sigma_w = 0.01$C/m^2 with increasing amount of coating with (CTAB cetyl trimethyl ammonium bromide), a cationic surfactant.

TABLE S1. **Examples of surface charge and point of zero charge for some solid-state nanopores materials.** A first possibility to get a neutral charged solid state nanopore is to use Silicon Nitride. The surface charge σ_w varies with the pH. The point of zero charge pH is around 4.1 as confirmed by several works by different groups, see, *e.g.* [S19, S20]. Silicon Nitride can also be functionalized allowing to get very weak charge (< 10 mC/m^2) at pH 7 [S21]. Another material used for nanopores is HfO_2 [S23], the point of zero charge pH of which is $\sim$ 7.5 [S22]. Functionalization may also be used on PET nanopores [S24] to tune the surface charge. A wide list of experimental works on pH-dependent surface charging and points of zero charge for several materials is reported in [S25]. Some of these materials are currently used in nanopore experiments.

SUPPLEMENTARY REFERENCES

[S1] P Läuger, W Lesslauer, E Marti, and J Richter. Electrical properties of bimolecular phospholipid membranes. *Biochimica et Biophysica Acta (BBA)-Biomembranes*, 135(1):20–32, 1967.

[S2] Reto B Schoch, Jongyoon Han, and Philippe Renaud. Transport phenomena in nanofluidics. *Reviews of modern physics*, 80(3):839, 2008.

[S3] Martin Z Bazant and Todd M Squires. Induced-charge electrokinetic phenomena. *Current Opinion in Colloid & Interface Science*, 15(3):203–213, 2010.

[S4] Yao Yao, Chenyu Wen, Ngan H Pham, and Shi-Li Zhang. On induced surface charge in solid-state nanopores. *Langmuir*, 36(30):8874–8882, 2020.

[S5] Henrik Bruus. *Theoretical microfluidics.* Oxford university press Oxford, 2008.

[S6] Nikita Kavokine, Roland R Netz, and Lydéric Bocquet. Fluids at the nanoscale: From continuum to subcontinuum transport. *Annual Review of Fluid Mechanics*, 53:377–410, 2020.

[S7] Mauro Chinappi and Fabio Cecconi. Protein sequencing via nanopore based devices: a nanofluidics perspective. *Journal of Physics: Condensed Matter*, 30(20):204002, 2018.

[S8] Emma Letizia Bonome, Fabio Cecconi, and Mauro Chinappi. Electroosmotic flow through an α-hemolysin nanopore. *Microfluidics and Nanofluidics*, 21(5):96, 2017.

[S9] Kherim Willems, Dino Ruić, Florian LR Lucas, Ujjal Barman, Niels Verellen, Johan Hofkens, Giovanni Maglia, and Pol Van Dorpe. Accurate modeling of a biological nanopore with an extended continuum framework. *Nanoscale*, 12(32):16775–16795, 2020.

[S10] James Wilson, Kumar Sarthak, Wei Si, Luyu Gao, and Aleksei Aksimentiev. Rapid and accurate determination of nanopore ionic current using a steric exclusion model. *ACS sensors*, 4(3):634–644, 2019.

[S11] William Humphrey, Andrew Dalke, and Klaus Schulten. Vmd: visual molecular dynamics. *Journal of molecular graphics*, 14(1):33–38, 1996.

[S12] Pauli Virtanen, Ralf Gommers, Travis E. Oliphant, Matt Haberland, Tyler Reddy, David Cournapeau, Evgeni Burovski, Pearu Peterson, Warren Weckesser, Jonathan Bright, Stéfan J. van der Walt, Matthew Brett, Joshua Wilson, K. Jarrod Millman, Nikolay Mayorov, Andrew R. J. Nelson, Eric Jones, Robert Kern, Eric Larson, CJ Carey, İlhan Polat, Yu Feng, Eric W. Moore, Jake Vand erPlas, Denis Laxalde, Josef Perktold, Robert Cimrman, Ian Henriksen, E. A. Quintero, Charles R Harris, Anne M. Archibald, Antônio H. Ribeiro, Fabian Pedregosa, Paul van Mulbregt, and SciPy 1. 0 Contributors. SciPy 1.0: Fundamental Algorithms for Scientific Computing in Python. *Nature Methods*, 17:261–272, 2020.

[S13] J. D. Hunter. Matplotlib: A 2d graphics environment. *Computing in Science & Engineering*, 9(3):90–95, 2007.

[S14] Douwe Jan Bonthuis, Stephan Gekle, and Roland R Netz. Dielectric profile of interfacial water and its effect on double-layer capacitance. *Physical review letters*, 107(16):166102, 2011.

[S15] Gabriele Raabe and Richard J Sadus. Molecular dynamics simulation of the dielectric constant of water: The effect of bond flexibility. *The Journal of chemical physics*, 134(23):234501, 2011.

[S16] Joost H Weijs, Antonin Marchand, Bruno Andreotti, Detlef Lohse, and Jacco H Snoeijer. Origin of line tension for a lennard-jones nanodroplet. *Physics of fluids*, 23(2):022001, 2011.

[S17] Daan Frenkel and Berend Smit. *Understanding molecular simulation: from algorithms to applications*, volume 1. Elsevier, 2001.

[S18] Peter Atkins and Julio De Paula. *Physical chemistry for the life sciences.* Oxford University Press, USA, 2011.

[S19] Kabin Lin, Zhongwu Li, Yi Tao, Kun Li, Haojie Yang, Jian Ma, Tie Li, Jingjie Sha, and Yunfei Chen. Surface charge density inside a silicon nitride nanopore. *Langmuir*, 37(35):10521–10528, 2021.

[S20] David P Hoogerheide, Slaven Garaj, and Jene A Golovchenko. Probing surface charge fluctuations with solid-state nanopores. *Physical review letters*, 102(25):256804, 2009.

[S21] YM Nuwan DY Bandara, Buddini I Karawdeniya, James T Hagan, Robert B Chevalier, and Jason R Dwyer. Chemically functionalizing controlled dielectric breakdown silicon nitride nanopores by direct photohydrosilylation. *ACS applied materials & interfaces*, 11(33):30411–30420, 2019.

[S22] M Kosmulski. Attempt to determine pristine points of zero charge of nb2o5, ta2o5, and hfo2. *Langmuir*, 13(23):6315–6320, 1997.

[S23] J Larkin, R Y Henley, M Muthukumar, J K Rosenstein, and M Wanunu. High-bandwidth protein analysis using solid-state nanopores. *Biophys. J.*, 106(3):696–704, 2014.

[S24] Yanbo Xie, Jianming Xue, Lin Wang, Xinwei Wang, Ke Jin, Long Chen, and Yugang Wang. Surface modification of single track-etched nanopores with surfactant ctab. *Langmuir*, 25(16):8870–8874, 2009.

[S25] Marek Kosmulski. The ph-dependent surface charging and the points of zero charge. *Journal of Colloid and Interface Science*, 253(1):77–87, 2002.
